# Defining super-enhancer landscape in triple-negative breast cancer by multiomic profiling

**DOI:** 10.1038/s41467-021-22445-0

**Published:** 2021-04-14

**Authors:** Hao Huang, Jianyang Hu, Alishba Maryam, Qinghua Huang, Yuchen Zhang, Saravanan Ramakrishnan, Jingyu Li, Haiying Ma, Victor W. S. Ma, Wah Cheuk, Grace Y. K. So, Wei Wang, William C. S. Cho, Liang Zhang, Kui Ming Chan, Xin Wang, Y. Rebecca Chin

**Affiliations:** 1 Department of Biomedical Sciences, City University of Hong Kong, Kowloon, Hong Kong; 2 Key Laboratory of Biochip Technology, Biotech and Health Centre, Shenzhen Research Institute, City University of Hong Kong, Shenzhen, China; 3 Department of Breast Surgery, The Affiliate Tumor Hospital, Guangxi Medical University, Nanning, China; 4 Department of Clinical Oncology, Queen Elizabeth Hospital, Kowloon, Hong Kong; 5 Department of Pathology, Queen Elizabeth Hospital, Kowloon, Hong Kong

**Keywords:** Breast cancer, Gene regulatory networks

## Abstract

Breast cancer is a heterogeneous disease, affecting over 3.5 million women worldwide, yet the functional role of cis-regulatory elements including super-enhancers in different breast cancer subtypes remains poorly characterized. Triple-negative breast cancer (TNBC) is an aggressive subtype of breast cancer with a poor prognosis. Here we apply integrated epigenomic and transcriptomic profiling to uncover super-enhancer heterogeneity between breast cancer subtypes, and provide clinically relevant biological insights towards TNBC. Using CRISPR/Cas9-mediated gene editing, we identify genes that are specifically regulated by TNBC-specific super-enhancers, including FOXC1 and MET, thereby unveiling a mechanism for specific overexpression of the key oncogenes in TNBC. We also identify ANLN as a TNBC-specific gene regulated by super-enhancer. Our studies reveal a TNBC-specific epigenomic landscape, contributing to the dysregulated oncogene expression in breast tumorigenesis.

## Abstract (web summary)

Triple-negative breast cancer (TNBC) is an aggressive breast cancer subtype with poor prognostic outcomes. Here the authors characterize super-enhancer heterogeneity and they identify genes that are specifically regulated by TNBC-specific super-enhancers, including FOXC1, MET and ANLN.

## Introduction

Breast cancer is the most common cancer and the leading cause of cancer death among women worldwide^1^. Molecular studies based on gene expression profiles have revealed different breast cancer subtypes: luminal, HER2+/ER−, and basal like. The majority of basal-like tumors (~70%) are triple-negative (ER-/PR-/HER2-)^2,3^. Chemotherapy, usually with high toxicity, is the main treatment option for triple-negative breast cancer (TNBC), thus underscoring the clinical need for identifying novel therapeutic targets for this aggressive subtype. Key genomic alterations associated with different subtypes of breast cancer, including PTEN, PIK3CA, HER2, BRCA, and TP53, have been comprehensively characterized. However, the lack of common genetic alternations in TNBC has limited the development of targeted therapies for this malignancy. On the other hand, the knowledge of deregulation of breast cancer epigenome in different subtypes that leads to various phenotypic outcomes remains poorly understood.

Transcriptome reprogramming is one of the critical features of cancer, where aberrant oncogene or tumor suppressor expression contributes to tumor initiation, progression, and metastasis. It can be due to genetic changes such as copy numbers, chromosomal rearrangement, and somatic mutations of protein-coding genes. Gene expression alteration could also be caused by cis-element changes in noncoding genomic regions where transcription factors (TFs) or other regulators bind to^4,5^. Alteration of these epigenomic marks has been shown to play important roles in the development of different diseases including cancers. For example, cryptic promoters with modified histone marks were identified to drive gastric malignancy^6^.

Enhancers are noncoding regions of the genome that harbor cis-regulatory elements and promote transcription of a target gene. They are characterized by histone modifications such as acetylation of histone H3 at lysine 27 (H3K27ac) and binding of coactivators such as p300^7^. Super-enhancers (SEs) are large clusters of enhancers that drive specific expression programs that define cellular identity^8^. They have been associated with different disease states such as the enrichment of enhancer clusters in pancreatic islet cells of Type 2 diabetes individuals^9^. It has been shown recently that SEs also play a critical role in upregulating the expression of cancer driver genes^10,11^. For example, focal amplifications of SEs that drive MYC expression have been identified in multiple epithelial cancers^7^. In gastric cancer, reprogramming and heterogeneity of SEs are observed during tumorigenesis, underpinning TF occupancy, and disease outcome^12^. Recently, CD47 and ERG are shown to be upregulated by SEs in breast and prostate cancer, respectively^13,14^. It has also been demonstrated that in neuroblastoma, expression of PRRX1 alters the SE and mRNA landscapes toward a mesenchymal state^15^. Epigenomic profiling has been done in various solid tumors, including medulloblastoma^16^ and colon cancer^17^, for identifying subgroup-specific SEs. However, comprehensive catalogs of SEs in different subtypes of breast cancer, and their functional importance in tumorigenesis, are not published yet.

In this study, we aim at characterizing the SE landscape in breast cancer. To dissect the heterogeneity of SEs in TNBC and non-TNBC subtypes, we perform integrative transcriptomic and epigenomic analyses, and provide evidence that SEs play an important role in characterizing subtype-specific identity. We also identify corresponding targeting genes of TNBC-specific SEs, including FOXC1, MET, and ANLN. The critical function of TF FOXC1 in breast cancer growth and its clinical relevance has been determined^18,19^. Phenotypically, overexpression of FOXC1 results in increased tumor growth and migration. However, very few studies have addressed the regulatory network of FOXC1. Here, our network biology analysis defines FOXC1 as the major node in regulating the expression of invasion and metastasis signature genes. Using CRISPR/Cas9 genomic editing, we delete the FOXC1-associated SE, and find a significant reduction of FOXC1 expression, as well as impairment of spheroid and clonogenic growth, consistent with its oncogenic properties. In addition, we identify ANLN as a TNBC-specific gene regulated by SE, contributing to TNBC tumorigenicity. Taken together, our multiomic analysis has revealed breast cancer subtype-specific SE regulatory mechanisms, and we have characterized the critical function of SEs in the specific oncogenic signaling of TNBC.

## Results

### A landscape of putative distal super-enhancers of breast cancer cell lines

Through high-throughput cis-regulatory element mapping, epigenomic dysregulation including SE heterogeneity, has emerged to play critical roles in the pathogenesis of various cancer types^12,16^. In breast cancer, it has been demonstrated that enhancer transcription can be used to improve the fidelity of functional enhancer identification^20^. However, the knowledge for subtype-dependent heterogeneity of breast cancer is lacking. Here, we collected the ChIP-seq data of 17 public breast cancer cell lines and two triple-negative (TN), immortalized breast cell lines (Supplementary Table 1), genome-widely identified and profiled the landscapes of SEs for different subtypes of breast cancer.

**Fig. 1:**
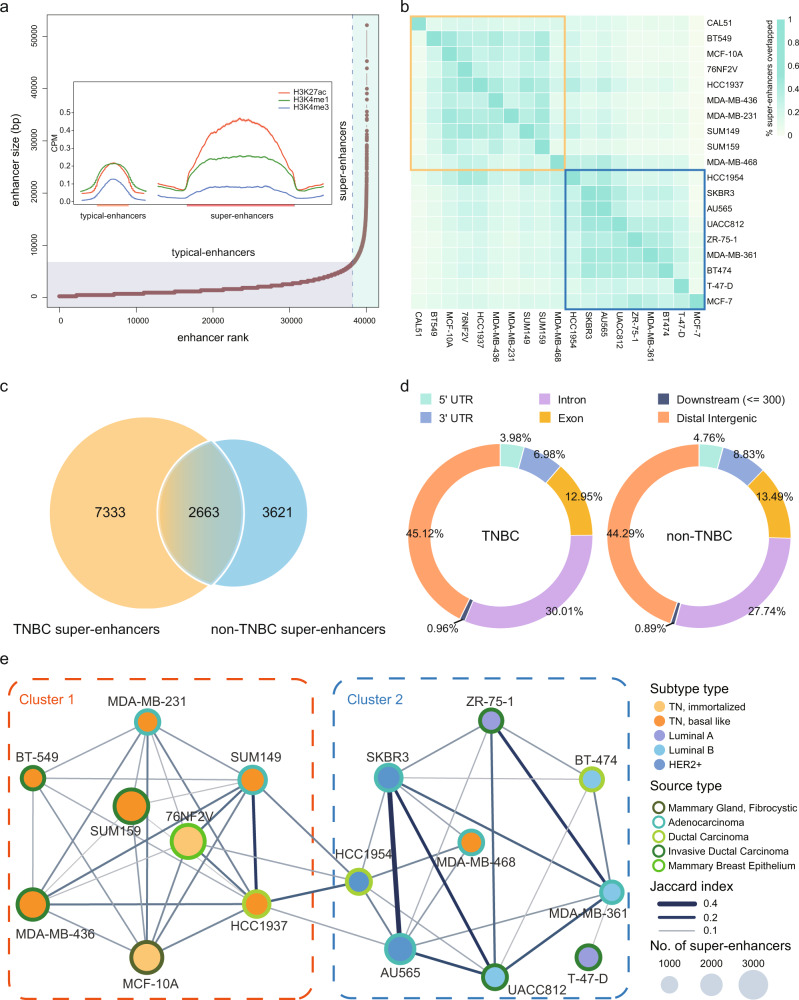
Epigenomic profiling identifies putative distal super-enhancers in breast cancer subtypes. **a** SEs were distinguished from typical enhancers using LOESS regression, fitting the size distribution of the enhancers followed by identification of the inflection point (slope = 1). Using AU565 cell line as an example, the identified SEs were enriched for higher H3K27ac and H3K4me1 signals, and lower H3K4me3 signals. **b** Cell lines belonging to the same subtype showed a higher degree of co-occurrence of SEs. The matrix shows pair-wise similarity of SEs detected in different cell types. The degree of similarity is colored in proportion to the overlap percentage. The top left part (orange rectangle) represents the TN cell lines, the bottom right part (blue rectangle) represents the non-TNBC cell lines. **c** Venn diagram shows the number of SEs uniquely found in at least one TNBC and non-TNBC cell lines, respectively, as well as those found both in TNBC and non-TNBC cell lines. **d** Genome-wide annotations of SEs detected in TNBC and non-TNBC cell lines, respectively. **e** A network of SE similarities between breast cancer cell lines. Each node represents a cell line and edge width corresponds to the significant Jaccard similarity coefficient (BH-adjusted *P* < 0.001, one-sided hypergeometric test). Based on network partition, two clusters of cell lines were identified, which recapitulates the subtype identify (TNBC vs. non-TNBC).

We annotated genome-wide cis-regulatory elements based on chromatin profiles identified by H3K27ac ChIP-seq signals, previously shown to mark active enhancers and other regulatory regions^21^. We focused on regions enriched with H3K27ac signals and located distant from transcriptional start sites (TSSs) of genes (up/downstream of 2.5 kb of TSSs), which were demonstrated by previous studies to be enhancer regions^12^. Next, SEs were identified from predicted active enhancers by fitting the size distribution of enhancers and identification of the inflection points (slope 1) using LOESS regression in the nineteen breast lines (Fig. 1a, Supplementary Table 2). For predicted SEs and typical enhancers, higher enrichment of H3K27ac and H3K4me1 signals (active markers in enhancer regions) and lower H3K4me3 signals (active markers in TSS) were observed, which were also consistent with the previous findings^22,23^. To explore the subtype-dependent heterogeneity of breast cancer, we first compared the degree of co-occurrence of SEs between TNBC and non-TNBC cell lines. We found that the average of within-cluster SE similarity scores (TNBC: 0.265; non-TNBC: 0.291) is significantly higher than the average of between-cluster similarity score (0.150) (both *P* < 0.001, Wilcoxon signed-rank tests), indicating that cell lines belong to the same subtype showed a higher degree of SE similarity (Fig. 1b). In addition, agreeing with previous gene expression profiling and enhancer studies^20^, the two TN, immortalized breast lines (MCF-10A, 76NF2V) show strong SE similarities with TNBC lines. Interestingly, TNBC cell line MDA-MB-468 showed a higher cross-subtype similarity with non-TNBC cell lines. By comparing the genome-wide loci of SEs and taking their union regions, we detected 6284 union-non-TNBC SEs and 9996 union-TNBC SEs, of which 7333 are TNBC specific (Fig. 1c). The distributions of TNBC and non-TNBC SEs in the genome are similar (Fig. 1d). The majority of SEs were located in the distal intergenic and intron regions, which patterns are also found in other tumor types^24,25^.

To further explore the heterogeneity of genome-wide SE profiles in different subtypes of breast cancer, we employed a network-based approach to investigate the association of 19 cell lines (Fig. 1e). More specifically, we built a network of SE similarities, where nodes represent cell lines, and weighted edges encoded Jaccard similarity coefficients quantifying the co-occurrence of SEs between cell lines. Using the cutoffs on the degree of SE co-occurrence (Jaccard similarity coefficient > 0.1) and statistical significance (BH-adjusted *P* < 0.001, Hypergeometric tests), only significant associations were retained to build the network (Supplementary Data 1). Markov cluster algorithm (MCL)^26,27^ was employed to detect the presence of network clusters that would indicate subtype patterns. Consequently, two clusters were detected based on SE properties: cluster 1 encompasses 6 TNBC lines and 2 TN, immortalized breast lines, whereas cluster 2 consists of 8 non-TNBC cell lines and 1 TNBC cell line. Because the intersection of the cell lines MCF7 and CAL51 with others was relatively small, and the fact that they did not meet the thresholding conditions of our network (Supplementary Data 1, Jaccard similarity coefficient > 0.1 and Hypergeometric test BH-adjusted *P* < 0.001), these two cell lines were excluded from the network and downstream analyses. Our network-based approach revealed that the clustering of SEs is sufficient to characterize the subtype identity (TNBC vs. non-TNBC), without prior knowledge of the transcriptomes or markers.

### Multiomic characterizations of TNBC-specific super-enhancers

**Fig. 2:**
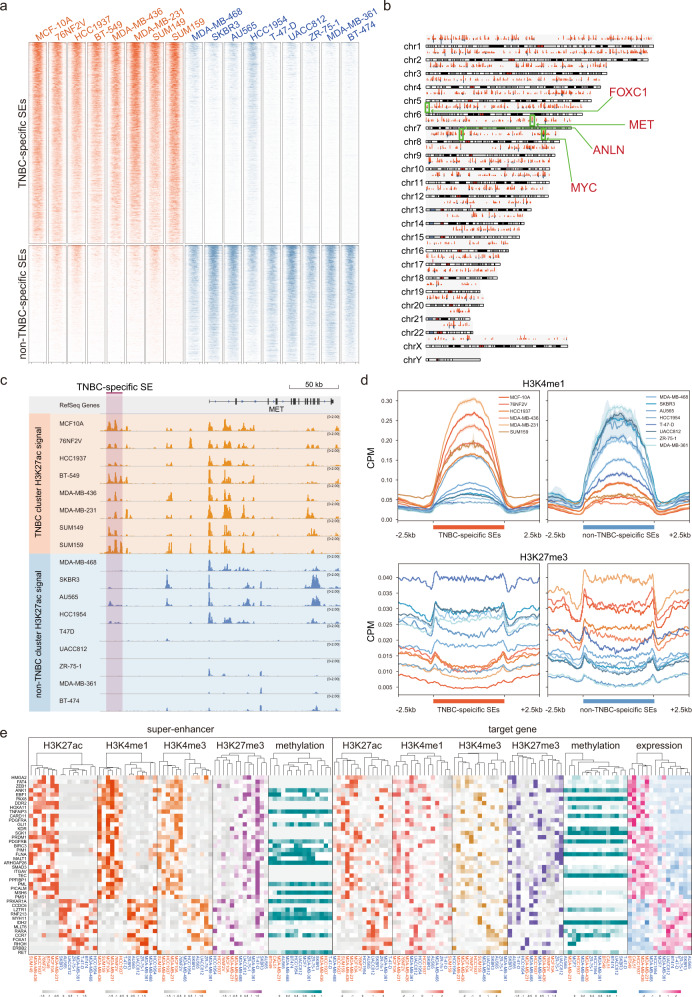
Multiomic characterization of subtype-specific super-enhancers. **a** Heatmap compares H3K27ac enrichment patterns between TNBC-specific SEs and non-TNBC-specific SEs across TNBC and non-TNBC cell lines. **b** A genome-wide overview of the loci and co-occurrence frequencies of identified TNBC-specific SEs in TNBC cell lines. Oncogenes FOXC1, MET and MYC were indicated. **c** Genome browser plot illustrates TNBC-specific SE detected in the upstream region of MET. **d** Higher enrichment of H3K4me1 signals was observed in TNBC-specific SEs in TNBC cell lines than non-TNBC cell lines, whereas H3K27me3 signals were depleted in TNBC-specific SEs in TNBC cell lines. **e** Heatmaps illustrate gene expression, DNA methylation, H3K27ac, H3K4me1, H3K4me3, H3K27me3 patterns of cancer-related genes in TNBC cell lines compared to non-TNBC cell lines. In each heatmap, the cell lines were organized by hierarchical clustering, with labels colored in orange and blue highlighting TN lines and non-TNBC lines, respectively.

For more comprehensive characterizations at multiomic levels, we focused on SEs that are significantly enriched or depleted for H3K27ac signal in TNBC cell lines compared to non-TNBC cell lines (|log2 fold enrichment|> 1 & BH-adjusted *P* < 0.05, Wilcoxon signed-rank tests). As a result, the 3035 TNBC-specific and 1765 non-TNBC-specific SEs showed distinct patterns of H3K27ac enrichment, and many oncogenes (e.g. FOXC1, MET, and MYC) are associated with the predicted TNBC-specific SEs (Fig. 2a, b). For example, MET, a receptor tyrosine kinase frequently altered in solid cancers^28^, was found to be located near a highly active SE in the TNBC lines (Fig. 2c).

As the subtype-specific SEs were detected based on H3K27ac signals, we next confirmed if other active or suppressive epigenetic markers also displayed different patterns in the subtype-specific SEs. As expected, signal of H3K4me1, a known active enhancer marker, was more highly enriched in the TNBC-specific SEs in the TNBC cell lines than the non-TNBC lines, and the opposite pattern was observed in the non-TNBC-specific SEs (Fig. 2d, Supplementary Fig. 1a, *P* < 0.001, Wilcoxon signed-rank test). On the other hand, H3K27me3 signal, known as a suppressive marker, was depleted in the TNBC-specific SEs in the TNBC cell lines (Fig. 2d, Supplementary Fig. 1b, *P* < 0.001, Wilcoxon signed-rank test). These data agree with histone modification profiles reported in previous studies^12,16^, and further confirmed the specificity of identified subtype-specific SEs.

Next, we integrated H3K27ac, H3K4me1, H3K4me3, H3K27me3 ChIP-seq data, DNA methylation and gene expression data to investigate potential associations in the SEs and corresponding genes (promoters). Using gene expression profiles of 15 cell lines obtained from CCLE, we specifically focused on cancer-related genes (obtained from Cancer Gene Census, https://cancer.sanger.ac.uk/census, |log2 fold change| > 0.5 & BH-adjusted *P* < 0.05) that were differentially expressed between the TNBC and non-TNBC cell lines, and predicted their potential regulatory SEs within 500 kb of upstream region. Consequently, we identified 29 upregulated cancer-related genes in TNBC cell lines and 13 upregulated cancer-related genes in non-TNBC cell lines. Using unsupervised classification of active markers, including H3K27ac, H3K4me1, and H3K4me3, in SEs or promoters of the target genes, we observed similar patterns across cell lines of the same subtype (Fig. 2e). As expected, the suppressive marker H3K27me3 was highly enriched in SEs and promoters of the non-TNBC cell lines. In contrast to histone modifications and gene expression, DNA methylation did not show clear differences between the TNBC and non-TNBC cell lines. These results highlight the dysregulation of cancer-related genes in TNBC governed by histone modifications and the implication of SEs in these processes.

### Integrative network analysis identified a SE-driven master regulator of invasion and metastasis in TNBC

Previous studies have shown that cancer cells acquired cancer-specific SEs at genes whose functions were associated with hallmark biological capabilities (or cancer hallmarks) during tumor development^10,29^. To elucidate the regulatory role of SEs in cancer hallmarks, we performed integrative analysis of the TNBC-specific SEs and gene expression profiles. Using the TCGA-BRCA dataset, we first identified 4501 genes differentially expressed (|log2 fold change | > 0.5 & BH-adjusted *P* < 0.05) between TNBC (n = 115) and non-TNBC samples (n = 605). To explore the functional relevance, we performed hypergeometric tests to evaluate overrepresentation of the differentially expressed genes in 10 cancer hallmark genesets collected from a previous study^10^. As a result, we identified three statistically significant genesets (BH-adjusted *P* < 0.05) associated with cancer hallmarks of “activating invasion and metastasis”, “sustaining proliferative signaling”, and “evading growth suppressors” (Supplementary Fig. 2a). To investigate whether SEs are involved in the regulation of genes in the dysregulated cancer hallmarks in TNBC, we looked for TNBC-specific SEs 500 kb upstream of genes upregulated in the TNBC samples. Consequently, we obtained 331 TNBC-specific SE-target-gene pairs (Supplementary Data 2), significantly associated with cancer hallmarks such as “evading growth suppressors” and “activating invasion and metastasis” (Supplementary Fig. 2b). For validation, we analyzed gene expression datasets of cell lines and nine independent cohorts of patient samples (Supplementary Table 1). Gene set enrichment analysis (GSEA) on these 10 datasets all confirmed the higher expression of these SE-target genes in TNBC (all *P* < 1 ×10^−4^, Supplementary Fig. 3–5).

**Fig. 3:**
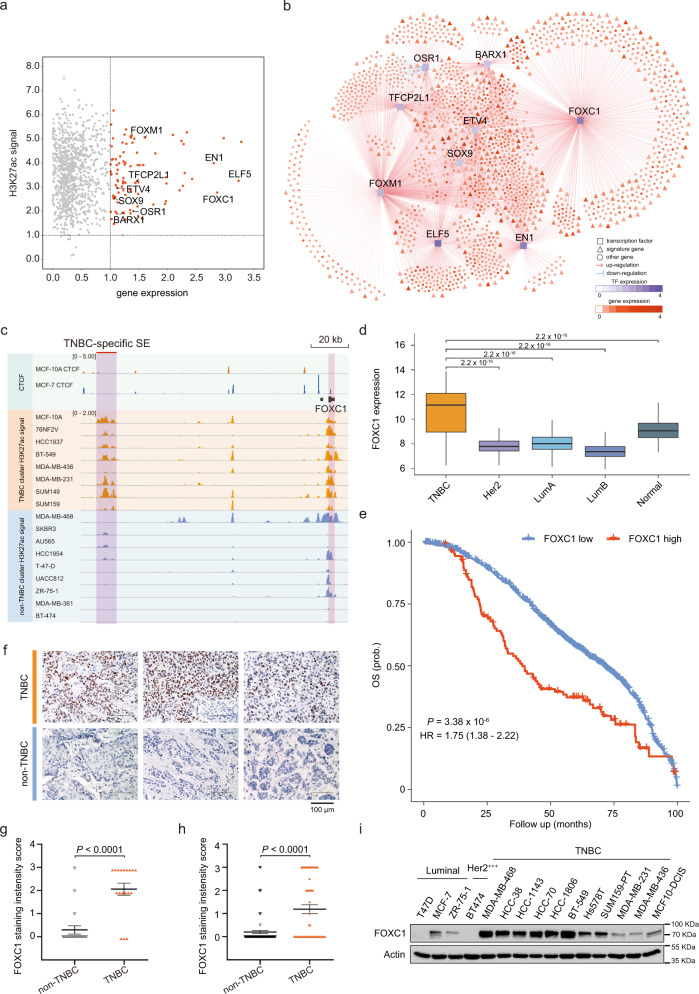
Identification of FOXC1 as a super-enhancer-driven master regulator of invasion and metastasis in TNBC. **a** Prediction of potential target genes of TNBC-specific SEs. The X-axis represents log2 fold change of gene expression between TNBC and non-TNBC samples in the TCGA-BRCA cohort. Y-axis represents log2 fold enrichment of H3K27ac signal of TNBC-specific SEs between TNBC and non-TNBC cell lines. Among all predicted target genes, nine transcription factors were identified and highlighted. **b** A regulatory network was inferred by integrative analysis of SE-regulated TFs and gene expression data in the TCGA-BRCA dataset. FOXC1 was identified as a master regulator of activating invasion and metastasis in TNBC. Predicted genes regulated by the nine TFs were colored in proportion to their differential expression levels between TNBC and non-TNBC samples. **c** Genome browser plot shows higher enrichment of H3K27ac signal in the SEs proximal to FOXC1 in the TNBC cell lines than the non-TNBC cell lines. **d** FOXC1 showed significantly higher expression in TNBC samples (*n* = 327) than those classified to the other subtypes (Her2 *n* = 237, LumA *n* = 706, LumB *n* = 484, and Normal-like *n* = 199) in the METABRIC cohort (two-sided Wilcoxon signed-rank tests). The boxes represent the 25th percentile, median, and 75th percentile, whiskers were extended to the furthest value that is no more than 1.5 times the inter-quartile range. **e** Kaplan–Meier plot shows that breast cancer patients with high expression of FOXC1 (top 10%) showed significantly poorer overall survival than the others in the METABRIC cohort. The statistical significance was calculated by a log-rank test (one-sided). (**f**, **g**) Immunohistochemistry of FOXC1 expression in 38 surgical breast cancer samples (18 TNBC, 20 non-TNBC). Representative FOXC1 staining images are shown. The bar graph shows the mean staining intensity score of FOXC1 in the surgical samples. (**h**) The mean staining intensity score of FOXC1 in tissue microarray samples of TNBC (*n* = 48) and non-TNBC (*n* = 102). **i** Immunoblotting detection of FOXC1 expression in a panel of breast cancer cell lines, repeated independently twice with similar results. Data are represented as mean ± SEM in (**g**) and (**h**). *P*-values were calculated by two-sided Student’s *t* test in (**g**) and (**h**). Source data are provided as a Source Data file.

It has been shown that the transcriptional program of TNBC is relatively uniform^30^, we thereby inferred a SE-based regulatory network using the TCGA-BRCA dataset to elucidate the regulation of cancer-related processes by TNBC-specific TFs. From the TNBC-specific SE-target genes, nine were identified as TFs (with log2 fold change > 1, Fig. 3a) and selected as potential regulators. 1785 upregulated genes in TNBC (log2 fold change > 0.5, BH-adjusted *P* < 0.05) were selected as potential target genes of the nine TFs. Integrating the gene expression levels of these TFs and potential target genes, we inferred a regulatory network based on the ARACNE algorithm^31–33^ (details in Methods) (Fig. 3b). To identify putative master regulators for TNBC, we performed master regulator analysis^33^, which tests for overrepresentation of each TF’s regulon for signature genes involved in a particular biological process. Using this strategy, we identified FOXC1 as the only statistically significant master regulator (BH-adjusted *P* < 0.05, a hypergeometric test, Supplementary Fig. 2c) of the cancer hallmark “activating invasion and metastasis” dysregulated in TNBC. FOXC1, belonging to the forkhead family of TFs which is characterized by a distinct DNA-binding forkhead domain, is associated with an activated TNBC-specific SE nearby (Fig. 3c). It was noted that a strong CTCF peak was observed right upstream of the promoter region of FOXC1 in non-TNBC-subtype cell line MCF-7 but not in TN, immortalized breast line MCF-10A, suggesting the potential insulator function in blocking the transcription of FOXC1 in non-TNBC. These results suggest that the subtype-dependent SE potentially regulates the expression of FOXC1, which plays a central role in the transcription reprogramming of invasion and metastasis in TNBC.

### TNBC-specific FOXC1 super-enhancer is required for tumor spheroid growth and invasion

**Fig. 4:**
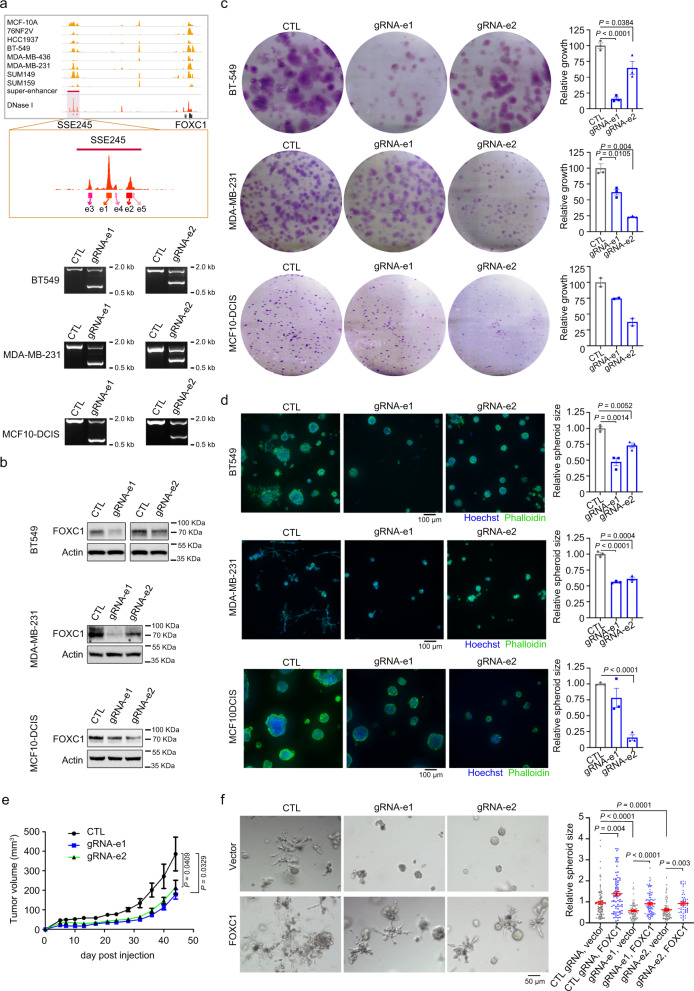
Deletion of FOXC1 SE reduces TNBC spheroid growth and invasion. **a** Schematic of FOXC1 SE (SSE245) and PCR detection of CRISPR/Cas9-mediated deletion of e1 and e2. Inset shows 5 constituent enhancers (e1–e5) within SSE245 and their sensitivities to DNase I in MDA-MB-231 cells. 1203 and 907 base pairs of e1 and e2 were deleted, respectively. **b** Immunoblotting of FOXC1 in BT549, MDA-MB-231, and MCF10-DCIS upon deletion of e1 or e2 of SSE245. Experiments in (**a**) and (**b**) were repeated twice independently with similar results. **c** Clonogenic assay of BT549, MDA-MB-231, and MCF10-DCIS with or without deletion of e1 or e2. Bar graphs show the quantification of clonogenic proliferation, *n* = 3 independent experiments for BT549 and MDA-MB-231, *n* = 2 for MCF10-DCIS. **d** Phalloidin and Hoechst staining of BT549, MDA-MB-231 and MCF10DCIS spheroids. Bar graphs show the quantification of spheroid size. *n* = 3 independent experiments. **e** Tumor volume of MDA-MB-231 xenografts with or without e1, e2 deletion. Tumor number of each group *n* = 7. **f** Growth of MDA-MB-231 spheroids with or without e1, e2 deletion in the presence of FOXC1 overexpression. Right panel, quantification of spheroid size. *n* = 98, 81, 87, 93, 99, 60 spheroids (from left to right) examined over 3 independent experiments. Data are represented as mean ± SEM in (**c**–**f**). *P*-values were calculated by two-sided Student’s *t* test in (**c**–**f**). Source data are provided as a Source Data file.

The clinical significance of FOXC1 in TNBC has been well established. Using an independent clinically annotated breast cancer gene expression dataset, we confirmed the significant upregulation of FOXC1 in TNBC compared to non-TNBC in METABRIC cohort (Fig. 3d), and higher expression of FOXC1 correlates with poor overall survival of breast cancer patients (Fig. 3e). These observations were further validated by immunohistochemistry in 38 FFPE surgical breast tumor samples and 150 samples on tissue microarray (Fig. 3f–h, Supplementary Table 3), in which FOXC1 upregulation in TNBC was significantly associated with higher grade (*P* = 9.72 × 10^−5^, Chi-square test), Ki67 (*P* = 6.14 × 10^−4^, Chi-square test) and tumor-infiltrating lymphocytes (*P* = 1.54 × 10^−6^, Chi-square test). Overexpression of FOXC1 was also observed in a panel of TNBC lines (Fig. 3i). The molecular mechanism by which this well-known oncogene is regulated, however, is poorly understood. DNase I hypersensitivity often coincides with enhancer activation. To determine experimentally if an SE mapping approximately 128 kb upstream of FOXC1 drives FOXC1 expression and tumorigenesis, we focused on two (e1, e2) of the five constituent enhancers within the SE region. e1 and e2 regions were chosen based on their greatest sensitivity to DNase I among the five constituent enhancers in TNBC cells (Fig. 4a). Using the CRISPR/Cas9 system, we deleted e1 or e2 in three TNBC lines, and verified deletion with PCR (Fig. 4a). Deletion of e1 or e2 resulted in marked decrease of FOXC1 expression in all three lines, demonstrating that FOXC1 is a target gene of the FOXC1 SE (Fig. 4b). Furthermore, deletion of e1 or e2 led to a significant reduction of clonogenic formation ability, spheroid size, and tumor growth (Fig. 4c–e), indicating the functional importance of FOXC1 SE in TNBC tumorigenicity. MDA-MB-231 cells exhibit an invasive phenotype in 3D culture, where invasiveness was reduced upon deletion of e1 or e2 (Fig. 4d), agreeing with the vital role of FOXC1 in TNBC invasion and metastasis (Fig. 3b)^34,35^. Indeed, knockdown of FOXC1 by shRNA resulted in morphology changes of TNBC cells, from spindle-shaped to cuboidal (Supplementary Fig. 6a, Supplementary Fig. 6b). Depletion of FOXC1 also significantly inhibited spheroid growth, accompanied with decreased invasive phenotype (Supplementary Fig. 6c). Furthermore, impaired clonogenic formation ability and xenograft growth were observed upon FOXC1 knockdown (Supplementary Fig. 6d, Supplementary Fig. 6e). Overexpression of FOXC1 promoted clonogenic growth of TNBC cells (Supplementary Fig. 6f). Importantly, inhibition of spheroid growth and invasiveness by FOXC1-SE deletion could be rescued by overexpression of FOXC1 (Fig. 4f, Supplementary Fig. 6g). These data point to the critical function of SE in upregulating FOXC1 for TNBC development.

**Fig. 5:**
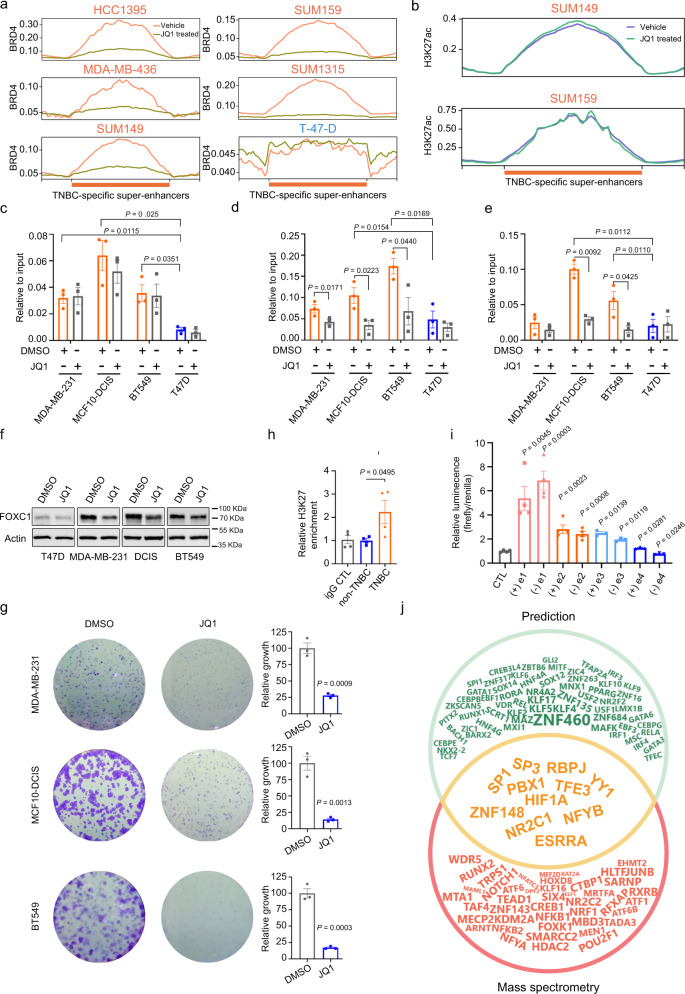
Epigenetic regulation of FOXC1 expression in TNBC. **a** ChIP-seq profiles for BRD4 in a panel of JQ1 or DMSO (vehicle control) treated breast tumor lines. **b** H3K27ac enrichment at TNBC-specific SEs with or without JQ1 treatment. **c–e** H3K27ac (**c**), Brd4 (**d**), and P300 (**e**) ChIP-qPCR of indicated cell lines using primer amplifying e1 of FOXC1 SE. *n* = 3 independent experiments. **f** Immunoblotting detection of FOXC1 expression in cells treated with JQ1 or DMSO, repeated independently twice with similar results. **g** Clonogenic growth of indicated cells with or without 0.3 µM JQ1 treatment. *n* = 3 independent experiments. **h** H3K27ac ChIP-qPCR of clinical breast cancer samples using primer amplifying e1 of FOXC1 SE. Four TNBC and four non-TNBC fresh frozen samples were tested. **i** Activity of constituent enhancers of FOXC1 SE measured in BT-549 cells by Dual-Luciferase reporter assay. *n* = 3 independent experiments. (**j**) Venn diagram shows TFs potentially binding to the SE region identified by prediction and mass spectrometry. The size of TFs uniquely found by prediction was proportionate to -log10 transformed *p*-value, and the size of TFs found by mass spectrometry only was in proportion to -log10 transformed BH-adjusted *p*-value. Data are represented as mean ± SEM in (**c**–**e**) and (**g**–**i**). *P*-values were calculated by two-sided Student’s *t* test in (**c**–**e**) and (**g**–**i**). Source data are provided as a Source Data file.

By targeting epigenetic regulation, BET inhibitors such as JQ1 have shown promising anticancer potential in various types of cancer including TNBC^36^. As a first step to characterize the effect of JQ1 on TNBC-specific SEs, ChIP-seq profiles for BRD4 in a panel of JQ1 or DMSO treated breast tumor lines were analyzed (Supplementary Table 1). JQ1 inhibits the binding of BRD4 to TNBC-specific SEs in TNBC lines (Fig. 5a). In contrast, there is a minimal binding of BRD4 to TNBC-specific SEs in the luminal line T47D, in the presence or absence of JQ1. As expected, JQ1 has no effect on the levels of H3K27ac in TNBC-specific SEs (Fig. 5b). To validate the enrichment of H3K27ac as well as the effect of JQ1 on binding of BRD4 and p300 on FOXC1 SE, we performed ChIP-qPCR on the SE region proximal to the FOXC1 gene. We observed significant enrichment of H3K27ac in e1 and e2 regions of TNBC but not luminal cells (Fig. 5c; Supplementary Fig. 7a). Furthermore, treatment of TNBC cells with JQ1 potently reduced the binding of BRD4 (Fig. 5d, Supplementary Fig. 7b) and p300 (Fig. 5e, Supplementary Fig. 7c) to the SE. As expected, H3K27ac levels were not affected by JQ1 (Fig. 5c). In line with the functional role of SE in regulating FOXC1 expression, JQ1 treatment reduced FOXC1 protein and mRNA levels in TNBC lines (Fig. 5f, Supplementary Fig. 7d). JQ1 treatment also significantly inhibited TNBC clonogenic and spheroid growth (Fig. 5g, Supplementary Fig. 7e). Extending these analyses to patient samples, we observed a significant enrichment of H3K27ac on the FOXC1 SE in TNBC samples as compared to non-TNBC tumors (Fig. 5h), agreeing with the upregulation of FOXC1 expression in the majority of TNBC cases (Figs. 3d, f, g, h). We then performed luciferase reporter assays for constituent enhancers e1–e4 (Fig. 4a). Agreeing with its greatest sensitivity to DNase I, e1 was found to have the strongest, orientation-independent activity (Fig. 5i), confirming it as the predominant regulatory element in the SE of FOXC1. Finally, we explored TFs associated with the constituent enhancer e1 of FOXC1 SE. Interrogating the nucleosome-free regions (NFR) defined by DNase-seq and known TF binding sites obtained from the JASPAR database (http://jaspar.genereg.net/)^37^, we discovered 96 candidate TFs binding to constituent enhancer e1 of SSE245 (Supplementary Data 3). We further performed DNA-pull-down assay followed by mass spectrometry analysis, and identified 55 candidate TFs binding to e1 (Supplementary Fig. 8, Supplementary Data 4), 11 of which overlapped with the ones predicted by TFs binding prediction analysis (Fig. 5j). Of the 11 candidates, eight and three TFs have been shown to be associated with transcription activating and repressing activities, respectively. Transcription activator HIF-1α has been reported to bind to FOXC1 promoter and enhance its expression in hepatocellular carcinoma^38^. Utilizing the ENCODE TF targets database, we also found SP1 and YY1 binding sites in FOXC1 promoter region. These three candidates will be prioritized for testing their role in SE-mediated overexpression of FOXC1 in future studies.

To further illustrate the feasibility of using our integrative approach to elucidate mechanisms by which oncogenes are specifically upregulated in TNBC, a parallel set of CRISPR/Cas9 studies was performed for MET, another oncogene highly expressed in TNBC and associated with worse clinical outcomes (Supplementary Fig. 9a–c). Similar to FOXC1, SE proximal to MET is enriched in H3K27ac and binding of Brd4 as well as p300 in TNBC lines (Supplementary Fig. 9d–f). In addition, JQ1 inhibited binding of Brd4 and p300 to the SE, and reduced MET expression in TNBC cells (Supplementary Fig. 9d–g). The functional significance of SE was demonstrated by the reduction of MET expression, colony formation, spheroid growth and invasiveness upon SE deletion in MDA-MB-231 and BT549 cells (Supplementary Fig. 9h–k). In MCF-10-DCIS cells, dCas9-Krab system^39^ was employed to inactivate the SE, which also led to reduced MET expression and colony forming ability (Supplementary Fig. 9l, m). These results substantiate our highly integrative approach in identifying TNBC-specific SEs and the oncogenes that they regulate.

### Cas9-mediated deletion of ANLN super-enhancer identifies its functional importance in TNBC clonogenicity

**Fig. 6:**
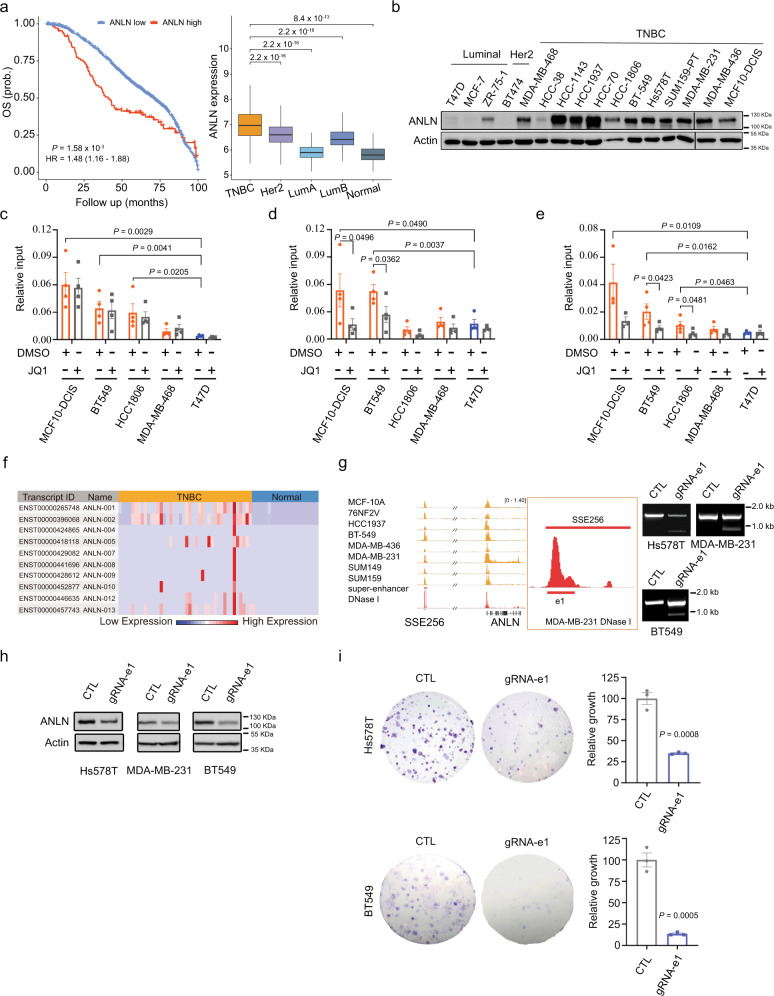
Functional importance of ANLN super-enhancer in TNBC clonogenicity. **a** Higher expression of ANLN is associated with poor prognosis of breast cancer patients in the METABRIC cohort. The statistical significance was calculated by a log-rank test (one-sided). Increased expression of ANLN in TNBC samples (*n* = 327), compared to other subtypes (Her2 *n* = 237, LumA *n* = 706, LumB *n* = 484, and Normal-like *n* = 199; two-sided Wilcoxon signed-rank tests). The boxes represent the 25th percentile, median, and 75th percentile, whiskers were extended to the furthest value that is no more than 1.5 times the inter-quartile range. **b** Immunoblotting detection of ANLN expression in a panel of breast cancer cell lines, repeated independently twice with similar results. **c**–**e** H3K27ac (**c**), Brd4 (**d**), and P300 (**e**) ChIP-qPCR of indicated cell lines using primer amplifying e1 of ANLN SE. *n* of independent experiments is indicated by scatter dots. **f** mRNA expression levels of ANLN, data from Cancer RNA-seq Nexus. TNBC *n* = 42 samples, Normal tissue *n* = 21 samples adjacent to TNBC. **g** Schematic of ANLN SE (SSE256) and detection of e1 deletion of ANLN SE by PCR in Hs578T, MDA-MB-231 and BT549 cells, repeated independently twice with similar results. **h** Immunoblotting detection of ANLN upon deletion of SSE256 e1 region, repeated independently twice with similar results. **i** Clonogenic assay of Hs578t and BT549 cells with or without deletion of SSE256 e1. *n* = 3 independent experiments. Data are represented as mean ± SEM in (**c**–**e**) and (**i**). *P*-values were calculated by one-sided Student’s *t* test in (**c**–**e**). *P*-values were calculated by two-sided Student’s *t* test in (**i**). Source data are provided as a Source Data file.

The functional role of FOXC1 and MET in TNBC has been well established. However, the biological significance of several other top hits from our SE target gene analysis (Supplementary Data 2) in TNBC remain elusive. To test if our integrative approach can also be applied to identify novel TNBC-specific genes driven by SEs, we focused on one of the top hits, ANLN, as its high expression in breast cancer patients has been shown to be significantly correlated to recurrence, expression of proliferation genes (all 17 tested in ref. ^40^), as well as poor overall survival (Fig. 6a;^41^). The specific function and regulation of ANLN in TNBC, however, are less investigated. The expression of ANLN was initially examined. Using clinically annotated breast cancer gene expression datasets, we found that the percentage of cases with ANLN mRNA upregulation is significantly higher in TNBC as compared to luminal breast tumors (Fig. 6a). In addition, in a panel of breast tumor lines, protein expression levels of ANLN are higher in TNBC lines as compared to luminal or Her2-overexpressed lines (Fig. 6b). This is in line with our bioinformatic prediction (Supplementary Data 2) and ChIP-PCR data (Fig. 6c–e) that ANLN is a potential target of SE enriched in TNBC. Importantly, ANLN is minimally expressed in normal breast tissues (42 TNBC tumors compared to 21 adjacent normal samples, http://syslab4.nchu.edu.tw/;^42^, Fig. 6f), suggesting the potential of targeting ANLN for therapeutic purposes. To investigate the functional role of ANLN SE, we used the CRISPR/Cas9 system to delete the peak of SSE256 (Fig. 6g). ANLN levels and clonogenicity were reduced significantly upon deletion of SE (Fig. 6h, i), indicating that the SE drives ANLN expression and TNBC progeny producing capability. Taken together, our results show that SE-based epigenomic characteristics can distinguish TNBC from non-TNBC subtypes, and identify TNBC-specific genes that determine the phenotypic outcomes. Our study identified a mechanism for upregulating FOXC1 and MET expression specifically in TNBC. Furthermore, our analysis uncovered ANLN as a TNBC-specific oncogene regulated by SE.

## Discussion

Although genetic abnormalities in breast cancer have been well studied and described, genomic-guided therapeutic strategies are lacking for TNBC patients. The high degree of genomic heterogeneity of TNBCs further limit the mutational-targeted therapies. Recent studies strongly suggest that deregulation of epigenome plays a crucial role in the pathogenesis of breast cancer^20,43^. However, how epigenetic changes influence subtype-specific SEs and the transcriptional program are not well understood. Our study was motivated by recent findings in other cancer types highlighting the significance of epigenetic circuitry, with the potential in yielding novel insights into mechanisms of tumorigenesis and identifying targets for therapeutic intervention. For example, subgroup-specific SEs have been identified for medulloblastoma^16^, providing a regulatory explanation for subgroups’ transcriptional diversity and clinical behaviors. In addition, chimeric TF PAX-FOXO1 was found to induce de novo SE in a specific subtype of rhabdomyosarcoma, thereby conferring selective therapeutic vulnerability to JQ1^23^. Although very little is known about SE in breast cancer, Franco et. al. has recently developed a computational pipeline to identify transcribed enhancers in different subtypes, and demonstrated that TFs including FOSL1 and PLAG1 play a key role in enhancer formation and the biology of TNBC cells^20^. Therapeutically, CDK7 inhibitors have been demonstrated to block tumor growth in patient-derived xenografts of TNBC, given the dependence of a cluster of key genes potentially driven by TNBC-specific SEs^30^. By performing ChIP-Seq and CRISPR-based screening, a recent study identified BAMBI as a SE-driven gene which regulates the growth of TNBC cells but not normal breast cells^44^. These studies underscore the importance of identifying and characterizing SEs to refine our understanding of epigenetic processes underlying TNBC tumorigenesis.

To obtain a genome-wide landscape of SEs in breast cancer, we performed ChIP-seq analysis for active enhancers (H3K27ac) in 19 established cell lines. Notably, unsupervised classification and network analysis discovered previously unrecognized SE heterogeneity in breast cancer, where genome-wide SE profiling is sufficient to characterize the subtype identity (TNBC vs. non-TNBC). We further identified 3035 TNBC-specific and 1765 non-TNBC-specific SEs and proved their specificity with multiple active and suppressive histone modification marks. By multiomic profiling of putative cancer-related genes with their corresponding regulatory SEs, we highlight the implication of SEs in governing the epigenetic regulation of TNBC pathogenesis via histone modifications instead of DNA methylation. One potential limitation of this analysis is the use of RRBS data covering only DNA methylation-enriched genome loci, which may not encompass the entire regions of SEs and promoters. Thus, it remains to be confirmed in the future if this finding is supported by DNA methylation data with a higher resolution (e.g., whole-genome bisulfite sequencing). Given the known relatively uniform transcriptional program of TNBC, we performed network biology analysis in breast cancer patient datasets to identify SE-driven TFs that act as master regulators of different cellular processes. Our analyses showed that a large proportion of TNBC-specific target genes are associated with multiple cancer hallmarks, and we were intrigued to uncover FOXC1 as the most significant regulator of activating invasion and metastasis. In this study, we predicted the SE target genes by searching for SEs within 500 kb upstream of genes that were upregulated in the TNBC samples. We further employed a public dataset (Supplementary Table 1) with Hi-C data for TN line MCF-10A and non-TNBC line MCF7 to investigate whether the SEs are looping to the promoters of their putative target genes. As the data are too sparse (MCF-10A: 169,655,013 contacts; MCF7: 165,433,519 contacts), we could not identify any significant loops across the entire genome. However, we found that contacts did exist between the SEs and the promoters of FOXC1 (Supplementary Fig. 10a), MET (Supplementary Fig. 10b) and ANLN (Supplementary Fig. 10c) in MCF-10A but not in MCF7, demonstrating that SE-promoter interactions are specific to TNBC.

Despite the well-studied functions and clinical significance of FOXC1 in breast cancer pathogenesis, little is known regarding its regulatory mechanisms. BMP4-SMAD signaling was shown to promote FOXC1 expression and osteogenic differentiation in myoblasts^45^, whereas canonical Wnt signaling was demonstrated to activate FOXC1 transcription in P19 embryonal carcinoma cells^46^. In basal-like breast cancer, p65 and GATA3 were shown to promote and inhibit FOXC1 expression, respectively^47,48^. In our study, to directly examine the functional role of SE in regulating FOXC1 expression, we deleted the associated SE by CRISPR/Cas9, and resulted in decreased expression of FOXC1 and TNBC spheroid growth and invasiveness, phenocopying downregulation of the gene. In clinical samples, we found H3K27ac enrichment at FOXC1 SE in TNBC tumors. We further showed that BRD4 and p300 enrichment at FOXC1 SE are sensitive to JQ1. Although JQ1 has recently shown efficacy in TNBC patients^36^, a mechanistic connection is yet to be demonstrated between BRD4 and SE. Our findings, therefore, not only illuminate the functional and clinical significance of TNBC-specific SEs, but also provide a mechanism for the specific overexpression of FOXC1 in TNBC. In lung adenocarcinoma and endometrial carcinoma, MYC overexpression was driven by focally amplified SE, which could be a potential common mechanism for upregulating oncogene expression in epithelial cancers^7^. We did not, however, observed FOXC1 SE focal amplification in our genomic analysis. To search for molecular mechanisms by which FOXC1 SE is selectively active in TNBC cells, we performed TFs binding prediction and mass spectrometry studies of enhancer e1 in SSE245, and identified 11 potential TFs associated with the FOXC1 SE. Among these TFs, HIF-1α has been implicated in the regulation of FOXC1 expression in solid tumors. In lung cancer, it was demonstrated that HIF-1α binds to the hypoxia-element in the FOXC1 promoter and drives FOXC1 transcription, resulted in enhanced tumor progression^49^. It would be interesting to test if HIF-1α mediates the SE-driven overexpression of FOXC1 in TNBC. Additionally, 162, 66, 45, and 39 TFs were also predicted to bind to the other four constituent enhancers e2, e3, e4, and e5 of SSE245, respectively (Supplementary Data 3). Among these TFs, FOXC1 was predicted to bind to two loci in e3, suggesting that FOXC1 potentially auto-regulates itself and interplays with other TFs as the core transcriptional regulatory circuitry in TNBC. Another central question relating to oncogenic SEs is how are they being acquired and formed. It has been demonstrated that tumor cells acquire SEs through a variety of mechanisms, including DNA mutation or indels at SE to generate de novo TF binding sites, chromosomal rearrangements, and changes in elements that define topologically associating domains^50^. In our study, we did not observe focal amplification of FOXC1 SE. The structural or DNA changes responsible for the formation of our newly identified TNBC-specific SEs remain to be investigated.

Through the multiomic analysis and deletion of SE peak, we identified ANLN, a gene involved in cytokinesis, as a target of TNBC-specific SE^51,52^. Unlike FOXC1 and MET, the functional role of ANLN in breast cancer has not been studied extensively. An association between ANLN expression and cancer susceptibility has been mainly inferred by gene expression studies. For example, the upregulation of ANLN is observed in various cancers including ovarian, lung, hepatic, pancreatic, colorectal, and breast cancer^41,53^. The oncogenic effect of ANLN has been attributed to its function in regulating cell cycle. In non-small lung cancer and breast cancer lines, cell proliferation is suppressed and large-sized polynucleated cells form upon ANLN depletion^41,54,55^. Recently, ANLN was shown to regulate breast cancer cell migration and stemness^56^. Given the migratory and invasive functions of FOXC1 and MET in breast cancer, it would be interesting to examine if there is coordinating upregulation of these three genes by SEs in driving the metastatic program of TNBC. Here, we identified a mechanism by which ANLN is upregulated in TNBC, these studies further suggest the clinical and biological significance of ANLN SE in TNBC tumorigenesis which warrants future investigations.

In summary, we have discovered distinct SE landscapes between TNBC and non-TNBC cells. Our studies highlight the functional significance of TNBC-specific SE in determining biological outcomes, and provide mechanisms by which key oncogenes specifically overexpressed in TNBC. We also demonstrate the utility of our multiomic platform in dissecting the regulatory circuits of gene expression and biology of different subtypes of breast cancer, further advocate for elucidating epigenetic dysregulation to provide insights for novel therapeutic interventions.

## Methods

### ChIP-seq data analysis

H3K27ac, H3K4me1, H3K4me3, H3K27me3, CTCF and BRD4 ChIP-seq reads were mapped to the human reference genome (UCSC hg19 genome build) using Bowtie (version 1.2.2)^57^, retaining only uniquely mapped reads for downstream analyses. For each mark, coverage tracks were generated using “deeptools” (version 3.3.0) and enrichment signals of histone modifications were scaled by Counts Per Million (or CPM) mapped reads^58^.

### Super-enhancer identification

For each cell type, H3K27ac enrichment regions were first identified using a two-state hidden Markov model (ChromHMM, version 1.17)^59^. H3K27ac enrichment regions located at gene promoters (within ±2.5 kb of TSS) were filtered out, retaining regions as predicted enhancers. For each cell line, super-enhancers were subsequently distinguished from typical enhancers based on LOESS regression by fitting the size distribution of the enhancers, followed by identification of the inflection point (slope 1).

### Association network analysis of super-enhancers

To study the associations between identified SEs in different cell lines, we employed a network-based approach. Each node in the association network encoded the number SEs in a cell line, and each edge encoded the Jaccard similarity coefficient calculated by the number of overlapped SEs between two cell lines over the size of their union. To quantify the statistical significance of SE associations between cell lines, hypergeometric tests for overrepresentation of SEs were performed. In the network, cell lines were connected if their SE associations are statistically significant (Benjamini–Hochberg (BH)-adjusted *P* < 0.001). Furthermore, MCL (Markov cluster algorithm) was employed to identify enriched modules of cell lines in the association network with the default parameter setting^26,27^.

### Subtype-specific super-enhancer identification

For each cell line, we first calculated the density of normalized mapped reads in SEs by Bamliquidator (version 1.3.8, https://github.com/BradnerLab/pipeline/wiki/bamliquidator)^16^. In brief, ChIP-seq reads of H3K27ac located within identified SEs with extended flanking regions (200 bp upstream and downstream) were used to calculate the density of reads per base pair, which was subsequently normalized by the total number of million mapped reads. The resulting read density in units of reads per million mapped reads per bp (rpm/bp) was defined as H3K27ac enrichment signals of SEs. For each SE, a Wilcoxon signed-rank test was used to assess whether H3K27ac enrichment signal is significantly differential between TNBC and non-TNBC cell lines. Log_2_ fold enrichment (or FE) of H3K27ac was calculated for each SE by the average signal in the TNBC cell lines over the counterpart in the non-TNBC cell lines. Differentially enriched SEs were defined by SEs with BH-adjusted *P* < 0.05 and |log_2_FE| > 1. More specifically, SEs with log_2_FE > 1 were identified as TNBC-specific SEs, and those with log_2_FE < −1 were non-TNBC specific.

### Reduced representation bisulfite sequencing data analysis

Reduced representation bisulfite sequencing (RRBS) raw reads were aligned to the human reference genome (UCSC hg19 assembly) using Bismark v0.20^60^, powered by bowtie2 (version 2.3.5)^61^, with default parameters. Based on the resulting BAM files, DNA methylation levels of SEs and promoter (±2.5 kb of TSS) regions of corresponding genes were calculated using the R package “methylKit” (version 1.2.10)^62^.

### Quantification of gene expression

The Cancer Genome Atlas (TCGA) level-3 gene expression data for 605 non-TNBC and 115 TNBC tissue samples (termed “TCGA-BRCA” dataset hereafter) were downloaded from Firehose Broad GDAC portal (https://gdac.broadinstitute.org/). The gene expression levels measured by RSEM (scaled estimates in the gene-level) were converted to TPM (transcripts per million) values by multiplying by 10^6^, followed by log2-transformation for the subsequent analyses. ‘limma’ (R package, version 3.32.2)^63^ was employed to identify genes that are significantly upregulated (log2 fold change > 0.5, BH-adjusted *P* < 0.05) in TNBC samples, compared to non-TNBC samples.

Fifteen cell lines in CCLE were analyzed in the project, for which RNA-seq raw data were downloaded from Sequence Read Archive (https://www.ncbi.nlm.nih.gov/sra, PRJNA523380). Sequencing reads were mapped to the human reference genome (UCSC hg19 assembly) using STAR (version 2.7.1a)^64^, where only uniquely mapped reads were retained for the subsequent analyses. Differential gene expression analysis was performed using the R package ‘DESeq2’ (version 1.26.0)^5^ between TNBC and non-TNBC cell lines.

### Prediction of potential TFs at SEs

MDA-MB-231 DNase-seq reads were aligned to the human reference genome (UCSC hg19 assembly) using Bowtie (version 1.2.2)^57^, retaining only uniquely mapped reads for downstream analyses. Binding peaks (*P* < 1 × 10^−5^) were identified using MACS software (version 2.1.0)^65^. Three DNase peaks, also called NFRs, were identified in SE of FOXC1 and further employed for identification of TFs that potentially bind to the SE. The potential binding sites for 746 TFs were detected at the NFRs using FIMO^66^ with default parameters (*P* < 1 × 10^−4^), using position frequency matrices from JASPAR database (http://jaspar.genereg.net/)^37^.

### Regulatory network inference and master regulator analysis

A regulatory network was inferred by integrative analysis of gene expression profiles of 720 breast cancer patient samples in the TCGA-BRCA dataset to investigate the relationships between SE-regulated TFs and potential targets. Nine TFs regulated by TNBC-specific SEs that are notably upregulated (H3K27ac log2 fold enrichment > 1, BH-adjusted *P* < 0.05 & gene expression log2 fold change > 1, BH-adjusted *P* < 0.05) in TNBC-subtype patients compared to the others, were identified as potential regulators. 1785 genes differentially expressed between TNBC and non-TNBC tumors (log2 fold change > 0.5, BH-adjusted *P* < 0.05) were considered as potential targets of the nine TFs. The gene expression data for the TFs and potential targets were integrated for network inference using the ‘RTN’ package (version 1.12.0)^32,33^. Master regulator analysis for a specific gene signature, cancer hallmark of ‘activating invasion and metastasis’, was performed with a hypergeometric test of overrepresentation of each TF’s predicted targets. FOXC1 was identified as the most statistically significant master regulator (BH-adjusted *P* < 0.05) in the TNBC subtype.

### Cell Culture

T47D, BT474, MCF-7, MDA-MB-436, MDA-MB-468, MDA-MB-231, Hs578T, ZR-75-1, HCC38, HCC1143, HCC70, HCC1806, BT-549 and HEK293T cells were obtained from ATCC. MCF10-DCIS and SUM159-PT cells were obtained from Kornelia Polyak (Harvard Medical School, USA). T47D, MCF-7, MDA-MB-468, MDA-MB-231 and HEK293T cells were maintained in Dulbecco’s modified Eagle medium (DMEM; Gibco) supplemented with 10% tet system-approved Fetal Bovine Serum (FBS; Clontech). ZR-75-1, HCC38, HCC1143, HCC70, HCC1806, BT-549 cells were cultured in RPMI 1640 medium (Gibco) supplemented with 10% FBS. BT474 was maintained in RPMI 1640 medium supplemented with 10% FBS and 10 µg/ml Insulin. Hs578T and MDA-MB-436 cells were maintained in DMEM supplemented with 10% FBS and 10 µg/ml Insulin. SUM159-PT cells were maintained in Ham’s F12 Medium (Lonza) supplemented with 5% FBS, 5 µg/ml Insulin and 500 ng/ml hydrocortisone. MCF10-DCIS cells^67^ were maintained in DMEM/F-12 (Gibco) supplemented with 5% horse serum, 20 ng/ml EGF, 10 μg/ml Insulin, 100 ng/ml cholera toxin and 500 ng/ml hydrocortisone. All cell lines obtained from the cell banks listed above were tested for authentication using short tandem repeat (STR) profiling and passaged for fewer than 6 months, and routinely assayed for *Mycoplasma* contamination.

### 3D cultures

3D cultures were prepared as previously described^68^. Briefly, 96-well plates were coated with growth factor-reduced Matrigel (BD Biosciences) and allowed to solidify for 30 min. Cells (2000–4000) in assay medium were seeded per well. Assay medium contained DMEM or RPMI 1640 supplemented with 10% FBS and 2% Matrigel. The assay medium was replaced every 3–4 days. After 6–7 days culture in 3D, spheroid number and cell number per spheroid were determined. Spheroids were fixed by 4% Formaldehyde for 20 min in room temperature and then permeabilized by 0.5%Triton-X/PBS for 3-4 min. Spheroids were then stained with 5.4 μM Hoechst 33342 and 0.165 μM AlexFluor-488 phalloidin for 45 min. CellInsight CX7 high-content screening platform (Thermofisher) was used to capture the signal of Hoechst 33342 and AlexFluor-488 phalloidin. 25 sequential fields per well were captured (×10 objectives). A z-stack range of 210 µm was acquired with a series of 35 z-slices separated by 6 µm. Maximum projection image of each z-stack was saved for spheroid number and cell number per spheroid analyses. To determined spheroid growth, CellTiter-Glo® 3D cell viability assay was performed according to the manufacturer’s protocol (Promega # G9682).

### Clonogenic growth assays

800 cells were seeded to 6-well plate and cultured for 12 days. Medium was changed every 4 days. After 12 days, cells were fixed with 4% formaldehyde for 15 min at room temperature. 0.1% crystal violet in ethanol was then used to stain colonies for 40 min followed by PBS wash. To quantify cell proliferation, wells were destained in 10% acetic acid, followed by reading the OD value of the samples at 595 nm with a spectrophotometer^7^.

### Antibodies

Anti-FOXC1 (#8758s) and anti-MET (#3127s) antibodies were obtained from Cell Signaling Technology. Anti-β-actin antibody was purchased from Sigma-Aldrich. Horseradish peroxidase-conjugated anti-mouse and anti-rabbit immunoglobulin G antibodies (AP307P, AP308P) were purchased from Millipore. For immunohistochemistry staining, anti-FOXC1 (# ab223850) antibody was purchased from Abcam. Anti-Brd4 (Bethyl Laboratories, #A301-985a100), anti-P300 (Bethyl Laboratories, #A300-358a), and anti-H3K27ac (Active motif, #39685) antibodies were used for Chip-qPCR. All primary and secondary antibodies for immunoblotting were used at 1:1000 and 1:5000 dilution, respectively. For immunohistochemistry, FOXC1 antibody was diluted at 1:500. For ChIP-qPCR, 3 µg of anti-H3K27ac was used per sample, 5 µg of anti-P300 and anti-BRD4 were used per sample.

### Plasmids

For deleting a peak of SE by CRISPR/Cas9 editing, a pair of gRNAs franking the peak were designed using http://crispr.mit.edu/. The pair of gRNAs (Supplementary Table 4) were then sequentially inserted into BsaI and BbsI restriction sites of px333 vector (Addgene 64073), which encodes spCas9 and 2 sgRNA cassettes. For deleting the SE peak with a lentiviral vector, FgH1tUTG (Addgene #70183) was used. The sequence of hU6 promoter-sgRNA-hU6 promoter-sgRNA was amplified by PCR and then cloned into PacI digested FgH1tUTG (to remove the H1t promoter and gRNA scaffold). shRNAs were cloned into pLKO.1 vector (Addgene 8453) or Tet-pLKO-puro (Addgene 21915) digested by AgeI and EcoRI. FOXC1 CDS was cloned into the CD532A-1 vector by Genewiz company.

### Transfection and lentiviral infection

To delete SE peak in MCF10-DCIS and BT549 cells, px333 was cotransfected with pBABE-puro vector using lipofectamine 3000 (Invitrogen). 48 h post-transfection, cells were treated with 1 µg/ml puromycin for 48 h, followed by collecting samples for PCR and other assays.

To produce lentiviral supernatants, 293 T cells were cotransfected with gRNA/Cas9-containing vectors, VSVG and psPAX2 for 60 h. Breast cancer cells were then infected with filtered lentiviral supernatants for 48–72 h.

### PCR

Genomic DNA was extracted using QIAamp DNA Mini Kit (Qiagen #51304). PCR was performed with Quick-Load^®^ Taq 2X Master Mix (NEB M0270L) using a Thermal Cycler (BioRad, C1000 Touch^TM^). Primers flanking the deleted region were designed using Primer-blast and synthesized by Integrated DNA Technologies company (Supplementary Table 4). PCR products were resolved on a 1.3% agarose gel.

### ChIP-qPCR

Cells were cross-linked with 1% PFA at room temperature for 5 min and then quenched with 125 mM glycine at room temperature for 5 min. Cells were washed twice with 1X TBS and harvested by scraping in 1 ml ChIP lysis buffer (50 mM HEPES, pH7.5, 140 mM NaCl, 1 mM EDTA, 1% Triton, 0.1% Na-deoxycholate, 1× proteinase inhibitor cocktail) and incubated on ice for 15 min. To shear the chromatin, cells were sonicated using Bioruptor Plus (Diagenode, UCD-300 TM) for 30 cycles (30 s ON and 30 s OFF at high power). Soluble chromatin was collected after two sequential high-speed centrifugations of the sonicated lysate (10,000*g* for 5 min and 15 min at 4 °C). 5% of the lysate was taken as input and the remaining lysate was incubated with specific antibodies at 4 °C for overnight. 30ul of pre-washed Protein G Sepharose (GE Healthcare, 17061802) were added to each sample and incubated at 4 °C for 1–2 h. The beads were washed with different buffers, once with ChIP lysis buffer, once with lysis buffer with 0.5 M NaCl, once with Tris/LiCl buffer (10 mM Tris, pH8.0, 0.25 M LiCl, 0.5% NP-40, 0.5% Na-deocycholate, 1 mM EDTA) and twice with Tris/EDTA buffer (50 mM Tris, pH8.0, 10 mM EDTA). After washing, 100ul of 10% chelex (Bio-Rad, cat. no. 142-1253) was added to the washed protein-G beads and boiled at 95 °C for 10 min and then 5 µl of 20 mg/ml Proteinase K (NEB, P8107S) were added and incubated at 37 °C for 30 min. Samples were boiled again for 10 min to inactivate proteinase K and centrifuged to collect the supernatant. 100 µl of 20 mM Tris, pH 8.0 was added to the pellet and centrifuged again to collect the supernatant. The supernatants were combined and it was used as a template for qPCR reaction. qPCR was performed using Applied Biosystems QuantStudio 3 Real-Time PCR System.

### Immunoblotting

Cells were lysed in EBC buffer (50 mM Tris-HCl, pH 7.4, 120 mM NaCl, 0.5% NP-40, 50 nM calyculin, 1 mM sodium pyrophosphate, 20 mM sodium fluoride, 2 mM EDTA, 2 mM EGTA) containing proteinase inhibitor cocktail. Protein concentration was determined using the Bio-Rad protein assay reagent. 20–40 µg lysates were separated by SDS-PAGE and detected with the indicated antibodies.

### Immunohistochemistry of clinical breast cancer samples

Breast tissue samples were obtained from breast cancer patients who underwent biopsy or surgery for resection at Queen Elizabeth Hospital. 5 µm formalin-fixed paraffin-embedded breast tissue sections were prepared, dewaxed in xylene, rehydrated in serial down-graded alcohols (100%, 95%, 70%) and brought down to water. Antigen retrieval was conducted by immersing the sections in pre-heated 90–95 °C Envision Flex Target Retrieval Solution of high pH (DAKO, Denmark, K8004) and microwaved for further 15 min. Sections were allowed to cool down in the retrieval solution for at least 20 min at room temperature. Rabbit anti-FOXC1 antibody (Abcam, USA, ab223850, dilution 1:500) was then applied on sections and incubated for 30 min at room temperature after blocking of endogenous peroxidase by EnVision FLEX Peroxidase-Blocking Reagent (DAKO, Denmark, SM801). Signal detection was facilitated by the addition of REAL EnVision Detection System and DAB chromogenic substrate (DAKO, Denmark, K5007) for 30 min and 2 min, respectively, at room temperature. Hematoxylin was used for nuclei counterstain. The stained sections were semi-quantitatively scored according to their staining intensity with negative/weak staining scored as grade 0/1, moderately strong staining scored as grade 2, and strong staining scored as grade 3. The procedures were approved by the Human Subjects Ethics Committees at City University of Hong Kong, and conformed to the government regulations for research involving human participants. Informed consent was obtained from the breast cancer patients.

### DNA-pull-down assay

DNA sequence of peak e1 of FOXC1 SE was synthesized by Ruibiotech company. Biotinated DNA sequence was generated by PCR using 5′-biotinated forward primer (Supplementary Table 4). PCR product was purified by QIAquick Gel extraction kit (Qiagen #28706). Cells were lysed in TNTE buffer (50 mM Tris/HCl (pH 7.6), 150 mM NaCl, 0.5% Triton X-100, 1 mM EDTA, protease and phosphatase inhibitors). Lysate was centrifuged at 13,000*g* for 10 min at 4 °C. Streptavidin-Agarose beads (Thermo Scientific) were washed with 1× B/W buffer (5 mM Tris-HCL (pH 7.5), 0.5 mM EDTA, 1 M NaCl), and then incubated with biotinylated PCR product in binding buffer (20 mM HEPES (pH 7.5), 2.5 mM KCl, 1 mM DTT, 20% glycerol, 0.02% NP40) for 30 min at room temperature, followed by wash once. Lysate was added to above prepared beads-biotinylated DNA complex, with or without 10 µg poly-dIdC(Sigma), in a total volume of 1.4 ml, and incubated for 6 h at 4 °C. For samples subjected to SDS-PAGE, complexes were washed 3 times with binding buffer. For samples subjected to mass spectrometry analysis, complexes were washed twice with binding buffer and then three times with 50 mM ammonium bicarbonate.

### Liquid chromatography with tandem mass spectrometry (LC-MS/MS)

Protein bands of interest were excised after DNA-pull down and SDS-PAGE. Bands were washed with 25 mM NH_4_HCO_3_/50% acetonitrile followed by dehydration with acetonitrile and reduction with 0.1 M NH_4_HCO_3_/10 mM TCEP solution at 55 °C for 45 min. Then samples were incubated with 0.1 M NH_4_HCO_3_/55 mM iodoacetamide solution at room temperature in dark for 45 min followed by wash, dehydration and dry by a vacuum centrifuge. For digestion, gel fragments were rehydrated by incubating with 20 ng/µl sequencing grade trypsin in 0.1 M NH_4_HCO_3_ on ice for 45 min followed by overnight incubation at 37 °C. Digested peptides were extracted and desalted using C18 tips (ThermoFisher, Cat# 87782) following manufacturer’s instruction and resuspended in 20 μL 0.1% FA buffer.

The LC-MS/MS analysis was done by an Easy-nLC 1200 system coupled to a Q Exactive HF mass spectrometry (Thermo Scientific). 6 µL of samples were injected into a reverse phase C18 column (Thermo Scientific Cat#164568) at a flow rate of 250 nL/min. A linear gradient of 7–25% of buffer B (0.1% formic acid in 80% acetonitrile) was run over a 50-min period by mixing mobile phase A (0.1% formic acid in ultrapure water) with eluting buffer mobile phase B. The electrospray voltage for ionization on the mass spectrometer was set at 2.3 kV. Positive ion mode was used for acquisition at a resolution of 120,000, with a full MS spectrum (m/z = 350-1800) using an automatic gain control (AGC) target of 3×10^6^. Top 12 most intense ions were selected for higher-energy collisional dissociation (HCD) fragmentation (normalized collision energy 27) and MS/MS spectra were generated with an AGC target of 1 × 10^5^ at a resolution of 30,000, with a dynamic exclusion time of 30 s.

All raw files produced by XCalibur 4.0.27 (Thermo Scientific) software were analyzed together using the Proteome Discoverer 2.2 software (ThermoFisher) against UniProt human protein database. The precursor and fragment mass tolerances were set to 10 ppm and 0.02 Da. A maximum of two missed cleavage sites of trypsin was allowed. Carbamidomethylation (C) was set as static modification. Oxidation (M) and acetyl (protein N-terminal) were set as dynamic modifications. False discovery rate (FDR) of peptide spectrum matches (PSMs) and peptide identification were determined using the Percolator algorithm at 1% based on *q*-value. For label-free quantification (LFQ), the Minora Feature Detector node in the processing workflow was used together with the Precursor Ions Quantifier node and the Feature Mapper node in the consensus workflow.

### Dual-Luciferase reporter assay

Fragments containing enhancer e1, e2, e3, or e4 of FOXC1-SE were first amplified from genomic DNA of BT-549 by PCR. The fragments obtained above were used as templates to amplified enhancer e1, e2, e3, and e4. Primers used for cloning are listed in Supplementary Table 4. Firefly luciferase reporter vector pGL3-Promoter (Promega, #E1761) was used for cloning of enhancer regions. The enhancers of FOXC1-SE were cloned upstream of the promoter-luc+ transcriptional unit of pGL3-Promoter vector using MluI and XhoI restriction enzyme sites. Enhancers were cloned in both orientations (+) and (-). The enhancer luciferase constructs were then cotransfected with pRL-TK vector (Promega, #E2241) into BT-549 cells using FuGENE 6 (Promega, #2693). pRL-TK vector was used as an internal control reporter vector. 48 h post-transfection, luminescence signal was read according to the manual of Dual-Glo^®^ Luciferase Assay System kit (Promega, #E2920). The firefly luciferase signal was first normalized to the Renilla luciferase signal and then normalized to the signal from the empty pGL3-promoter plasmid.

### Xenograft studies

Female nude mice (6–8-week old) were purchased from the laboratory animal services center, Chinese University of Hong Kong. All procedures were approved by the Animal Ethics Committees at City University of Hong Kong, and conformed to the government guidelines for the care and maintenance of laboratory animals. Mice were housed in room temperature of 20–24 °C, with humidity of 30–70% and light/dark cycle of 12 h/12 h. 5×10^6^ cells resuspended in 50% Matrigel were injected into mammary fat pats (MFPs) of the female nude mice. Tumor size was examined every 4 days for the duration of the experiment. Tumor volumes were calculated using the formula *V* = (*W*2 × *L*)/2, where *V* is the tumor volume, W is the tumor width, and *L* is the tumor length. To knockdown FOXC1 expression in MDA-MB-231 tumor, tet-on-pLKO-puro vector was used and drinking water containing 20 g/L sucrose and 1 g/L doxycycline was administered to induced the expression of FOXC1 shRNA. Drinking water was changed every week.

### Statistical analysis

Wilcoxon signed-rank tests were used to compare FOXC1 expression levels and H3K27ac enrichment signals between TNBC and non-TNBC cell lines. The Benjamini-Hochberg method was used to correct for multiple hypotheses testing. Hypergeometric tests for overrepresentation were performed to identify functional genesets associated with genes by R package ‘HTSanalyzeR2’^69^. Kaplan–Meier analysis with a log-rank test was performed to evaluate the statistical association between predicted target gene expression and overall survival. Statistical significance of correlation between FOXC1 expression levels and clinicopathology parameters was evaluated by Chi-square tests. All statistical analyses were performed using R (version 3.4.3, www.r-project.org).

### Reporting summary

Further information on research design is available in the Nature Research Reporting Summary linked to this article.

## Supplementary information

Supplementary Information

Description of Additional Supplementary Files

Supplementary Data 1

Supplementary Data 2

Supplementary Data 3

Supplementary Data 4

Reporting Summary

## Data Availability

ChIP-seq data (with corresponding input data) for H3K27ac (19 cell lines), H3K4me1 (15 cell lines), H3K4me3 (15 cell lines), H3K27me3 (14 cell lines), CTCF (2 cell lines) and BRD4 (5 cell lines, before and after JQ1 treated) markers, DNase-seq (2 cell lines) used in this study are available in Gene Expression Omnibus (GEO) and EMBL-EBI ArrayExpress under accession code GSE69107, GSE38548, GSE85158, GSE63584, GSE87424, GSE46073, GSE26831, GSE80592, GSE63109, GSE84579, GSE98551, GSE70764 and PRJEB9547. RNA-seq and reduced representation bisulfite sequencing (RRBS) data of 15 cell lines used in this study are available in EMBL-EBI ArrayExpress under accession code PRJNA523380. Hi-C data of MCF-10A and MCF-7 used in this study are available in GEO under accession code GSE66733. Gene expression profiles for primary breast cancer samples, together with molecular subtyping labels and corresponding clinical information used in this study are available in Firehose Broad GDAC portal (https://gdac.broadinstitute.org/, *n* = 720), Molecular Taxonomy of Breast Cancer International Consortium (METABRIC, https://www.cbioportal.org/, *n* = 1953) and GEO under accession code GSE5327, GSE1456, GSE2034, GSE2990, GSE11121, GSE3494, GSE7390, and GSE12276. Mass spectrometry data generated in this study is available in iPRoX, with project ID: IPX0002865000 and PXD number: PXD024505. More details about the data availability and curation were summarized in Supplementary Table 1. Source data are available as a Source Data file. The remaining data are available within the Article, Supplementary Information or available from the authors upon request. Source data are provided with this paper.

## Source data

Source data

## Code availability

The code used for the entire data analysis process is freely available on github (https://github.com/CityUHK-CompBio/TNBCSE_code).
