# Supplementary material for: Defining super-enhancer landscape in triple-negative breast cancer by multiomic profiling
Source: Nat Commun. 2021 Apr 14;12:2242. doi: 10.1038/s41467-021-22445-0 (PMC8046763; doi:10.1038/s41467-021-22445-0)
Supplement: Supplementary file 1 — Supplementary Information [file 41467_2021_22445_MOESM1_ESM.pdf]

**Supplementary Information**

# Defining super-enhancer landscape in triple-negative breast cancer by multiomic profiling

Hao Huang[1#], Jianyang Hu[1,2#], Alishba Maryam[1], Qinghua Huang[3], Yuchen Zhang[1], Saravanan Ramakrishnan[1], Jingyu Li[1,2], Haiying Ma[1,2], Victor WS Ma[4], Wah Cheuk[5], Grace YK So[5], Wei Wang[1], William CS Cho[4], Liang Zhang[1,2], Kui Ming Chan[1,2], Xin Wang[1,2*], Y Rebecca Chin[1,2*]

[1] Department of Biomedical Sciences, City University of Hong Kong, Hong Kong.

[2] Key Laboratory of Biochip Technology, Biotech and Health Centre, Shenzhen Research Institute, City University of Hong Kong, China.

[3] Department of Breast Surgery, The Affiliate Tumor Hospital, Guangxi Medical University, China.

[4] Department of Clinical Oncology, Queen Elizabeth Hospital, Hong Kong.

[5] Department of Pathology, Queen Elizabeth Hospital, Hong Kong.

*Correspondence to R.C. (rebecca.chin@cityu.edu.hk) and X.W. (xin.wang@cityu.edu.hk)

[#] These authors contributed equally to this work

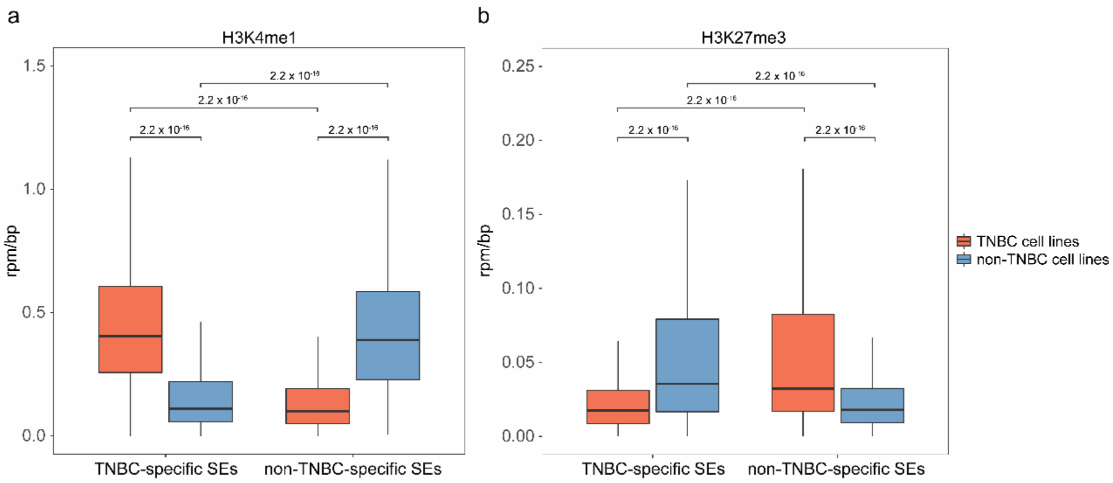


**Supplementary Fig. 1. Comparison of H3K4me1 signals and H3K27me3 signals between TNBC SEs and non-TNBC SEs in cell lines. (a)** Higher enrichment of H3K4me1 signals were observed in the TNBC-specific SEs (*n* = 3035) in six TNBC lines than eight non-TNBC lines, and the opposite pattern was observed in the non-TNBC-specific SEs (*n* = 1765), **(b)** H3K27me3 signals were significantly lower in the TNBC-specific SEs (*n* = 3035) in six TNBC lines than eight non-TNBC lines, and the opposite pattern was observed in the non-TNBC-specific SEs (*n* = 1765). *P*-values were calculated based on two-sided Wilcoxon signed-rank tests. The boxes represent the 25th percentile, median, and 75th percentile, whiskers were extended to the furthest value that is no more than 1.5 times the inter-quartile range.

a

b

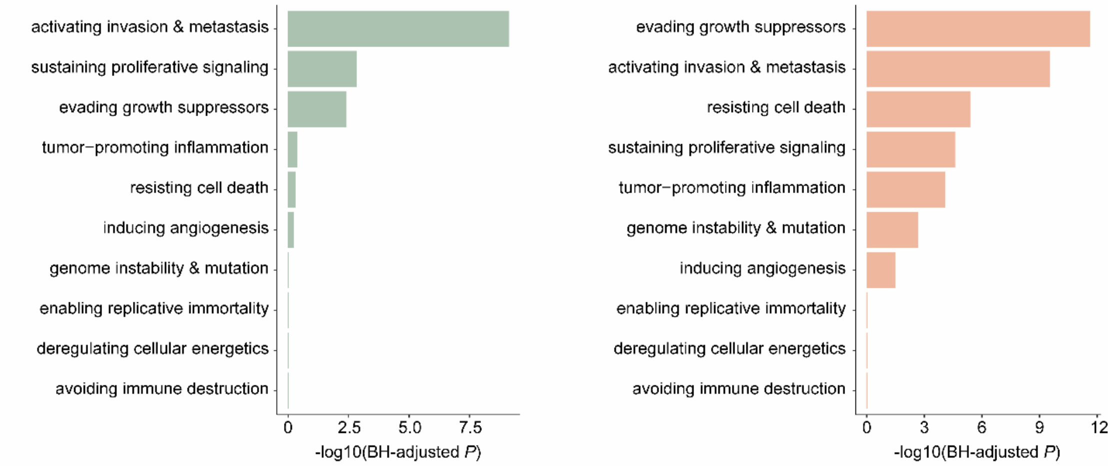


c

| regulator | universe size | regulon size | total hits | expected hits | observed hits | *P* | BH-adjusted *P* |
|---|---|---|---|---|---|---|---|
| **FOXC1** | **1785** | **754** | **302** | **127.57** | **171** | **2.45e-08** | **2.20e-07** |
| SOX9 | 1785 | 144 | 302 | 24.36 | 30 | 0.12 | 0.43 |
| EN1 | 1785 | 308 | 302 | 52.11 | 59 | 0.14 | 0.43 |
| BARX1 | 1785 | 163 | 302 | 27.58 | 28 | 0.49 | 1.00 |
| OSR1 | 1785 | 167 | 302 | 28.25 | 27 | 0.64 | 1.00 |
| FOXM1 | 1785 | 237 | 302 | 40.10 | 25 | 0.99 | 1.00 |
| OSR1 | 1785 | 194 | 302 | 32.82 | 221 | 0.99 | 1.00 |
| SOX9 | 1785 | 911 | 302 | 154.13 | 106 | 1.00 | 1.00 |
| TFCP2L1 | 1785 | 240 | 302 | 40.61 | 23 | 1.00 | 1.00 |

**Supplementary Fig. 2. Identification of FOXC1 as a master regulator of TNBC-related hallmark invasion and metastasis. (a)** Overrepresentation of differentially expressed genes (|log2 fold change| > 0.5 & BH-adjusted $P < 0.05$) between TNBC and non-TNBC patient samples in 10 cancer hallmark gene sets, ranked by the statistical significance (-log10(BH-adjusted *P*)), quantified by one-sided hypergeometric tests. **(b)** Overrepresentation of predicted TNBC-specific super-enhancer target genes in the 10 cancer hallmark gene sets, quantified by one-sided hypergeometric tests. **(c)** Master regulator analysis identified FOXC1 (highlighted in bold) as a master regulator regulating activating invasion and metastasis (BH-adjusted $P < 0.05$, one-sided hypergeometric test).

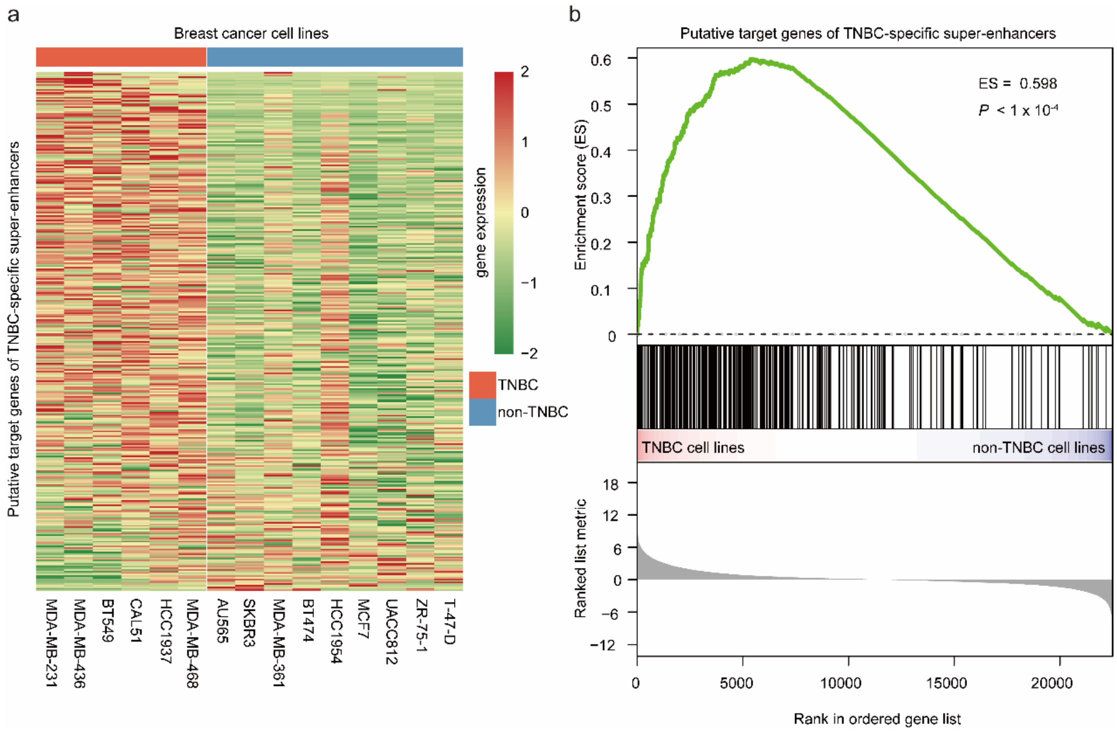


**Supplementary Fig. 3. The higher expression of the putative target genes of the TNBC-specific SEs in TNBC cell lines. (a)** Heatmap comparing the expression levels of putative target genes of TNBC-specific SEs between TNBC and non-TNBC cell lines. **(b)** GSEA confirmed that the putative target genes of TNBC-specific SEs are upregulated in TNBC cell lines ($P < 1 \times 10^{-4}$, 10,000 times permutation test).

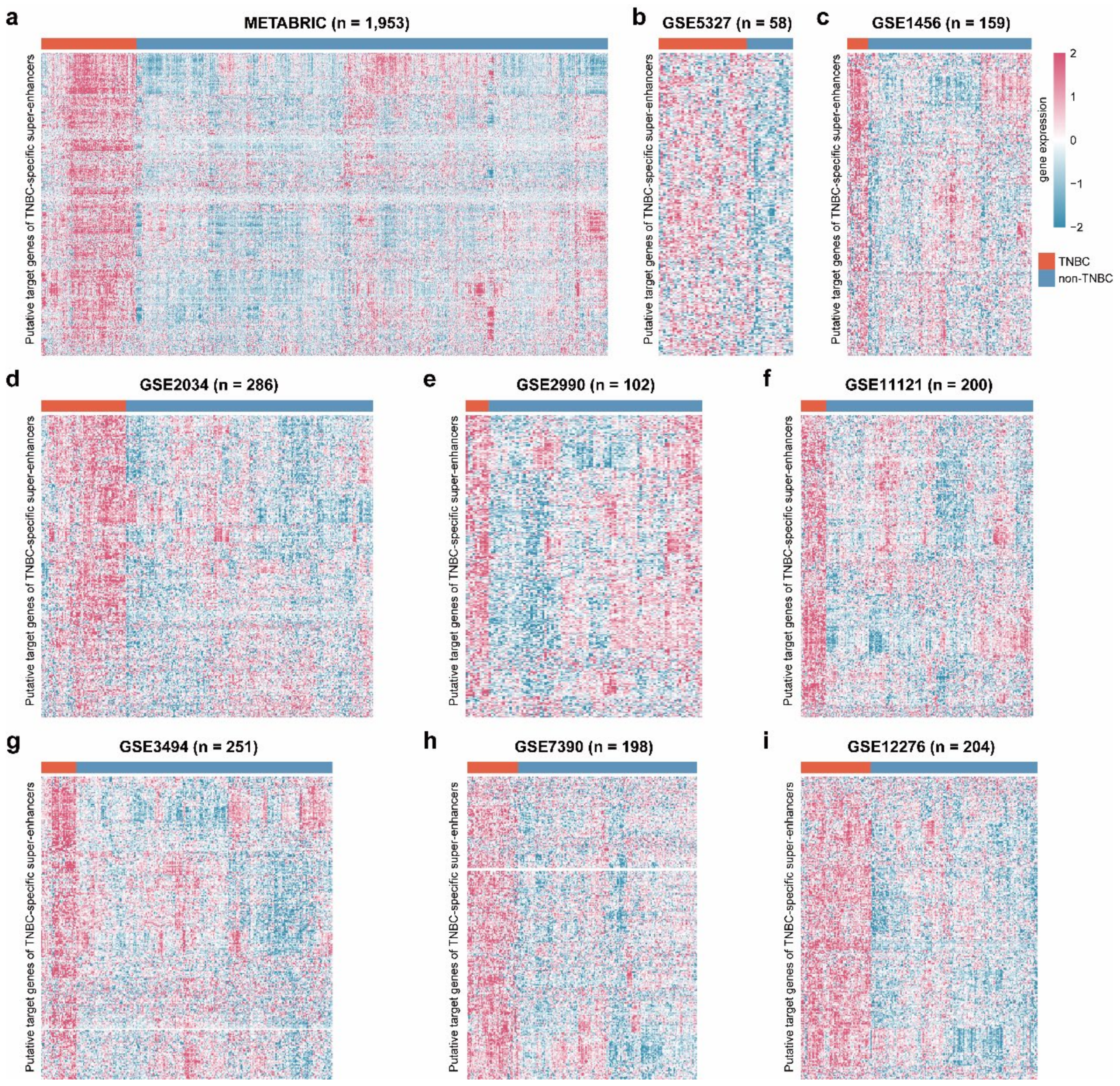


**Supplementary Fig. 4. The expression levels of the putative SE target genes in patient samples from nine independent cohorts.** Heatmap comparing the expression of putative target genes of TNBC-specific SEs between TNBC and non-TNBC patient samples in nine independent cohorts: **(a)** METABRIC (*n* = 1,953), **(b)** GSE5327 (*n* = 58), **(c)** GSE1456 (*n* = 159), **(d)** GSE2034 (*n* = 286), **(e)** GSE2990 (*n* = 102), **(f)** GSE11121 (*n* = 200), **(g)** GSE3494 (*n* = 251), **(h)** GSE7390 (*n* = 198), **(i)** GSE12276 (*n* = 204).

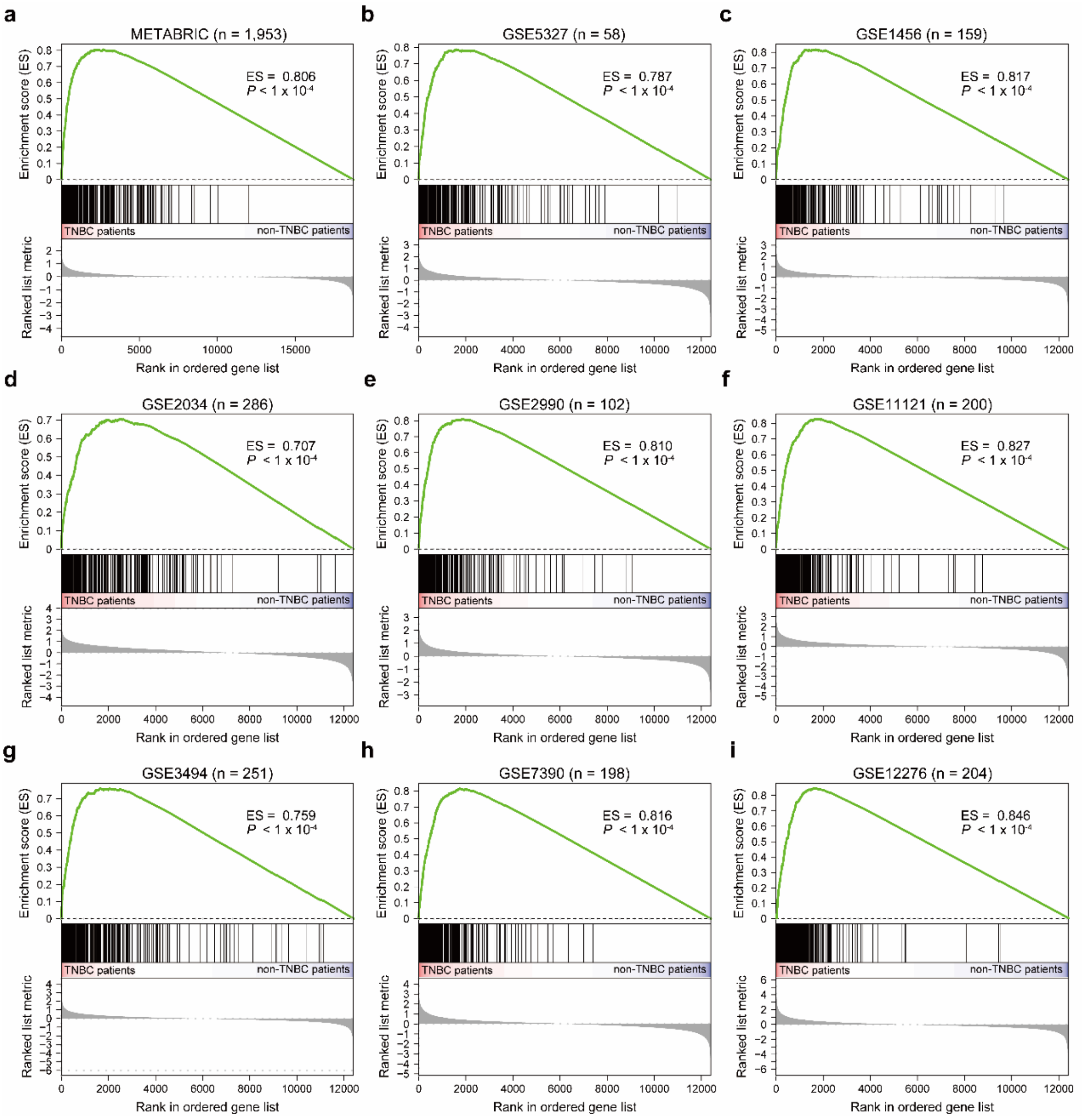


**Supplementary Fig. 5. TNBC-specific SE target genes are more highly expressed in TNBC patients.** GSEA confirmed that putative target genes of TNBC-specific SEs are upregulated in TNBC patient samples in the nine independent cohorts: **(a)** METABRIC (*n* = 1,953), **(b)** GSE5327 (*n* = 58), **(c)** GSE1456 (*n* = 159), **(d)** GSE2034 (*n* = 286), **(e)** GSE2990 (*n* = 102), **(f)** GSE11121 (*n* = 200), **(g)** GSE3494 (*n* = 251), **(h)** GSE7390 (*n* = 198), **(i)** GSE12276 (*n* = 204), quantified by 10,000 times permutation test.

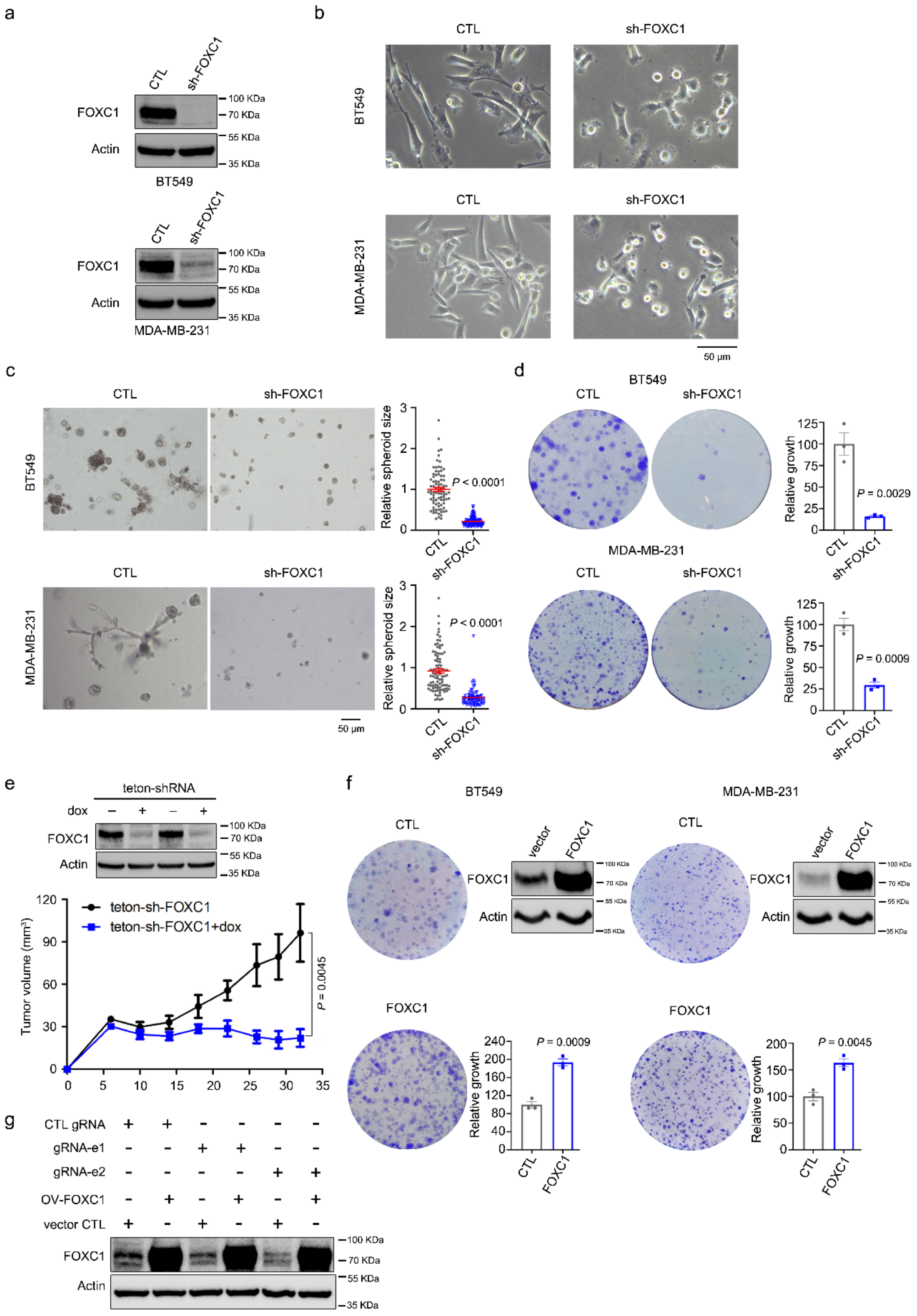


**Supplementary Fig. 6. Decreased TNBC growth by FOXC1 knockdown. (a)** Immunoblot of FOXC1 of BT549 and MDA-MB-231 cells upon FOXC1 knockdown. **(b)** Morphology of indicated cells upon FOXC1 knockdown. Experiments in **a** and **b** were

repeated twice independently with similar results. **(c)** BT549 and MDA-MB-231 spheroid growth upon FOXC1 knockdown. Right panel, quantification of spheroid size. *n* = 86 (CTL), 108 (sh-FOXC1) of BT549 spheroids and 93 (CTL), 77 (sh-FOXC1) of MDA-MB-231 spheroids examined over 3 independent experiments. **(d)** BT549 and MDA-MB-231 clonogenic growth upon FOXC1 knockdown. Right panel, quantification of clonogenic growth. *n* = 3 independent experiments. **(e)** FOXC1 shRNA was cloned in teton-pLKO-puro and knockdown in MDA-MB-231 cells was induced by doxycycline (dox) treatment. Immunoblotting shows knockdown of FOXC1 by teton-shRNA, repeated twice with similar results. Tumor volume of MDA-MB-231 xenografts with or without FOXC1 knockdown. Tumor number of each group *n* = 7. **(f)** BT549 and MDA-MB-231 clonogenic growth upon FOXC1 overexpression. *n* = 3 independent experiments. Immunoblotting was repeated independently twice with similar results. **(g)** Overexpression of FOXC1 in MDA-MB-231 cells with e1 or e2 deletion. Data are represented as mean ± SEM in **c**, **d**, **e** and **f**. *P*-values calculated by two-sided Student's *t*-test are indicated in **c**, **d**, **e** and **f**. Source data are provided as a Source Data file.

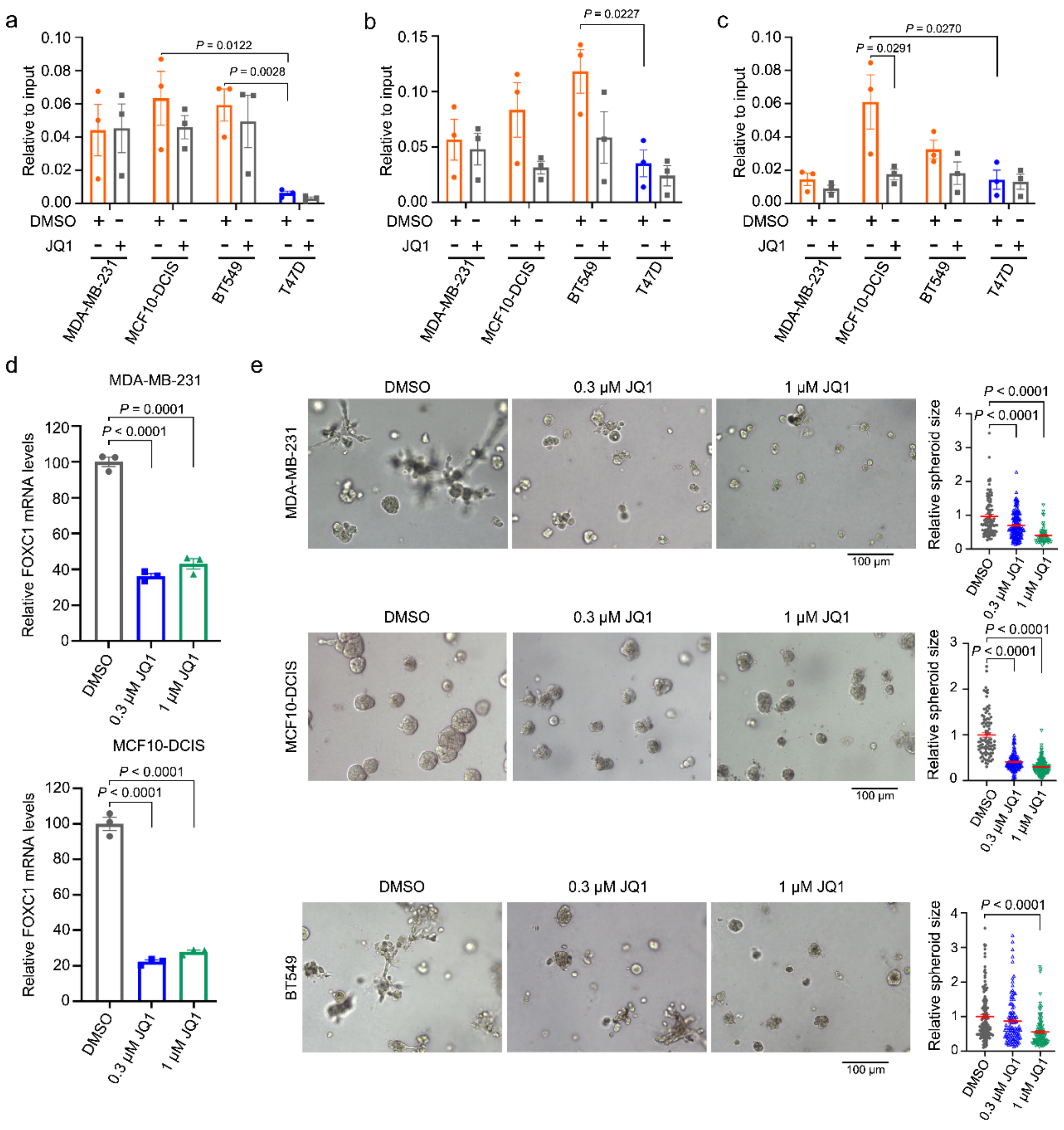


**Supplementary Fig. 7. The effect of JQ1 on FOXC1 SE.** H3K27ac **(a)**, Brd4 **(b)** and P300 **(c)** ChIP-qPCR of indicated cell lines using primers amplifying e2 of FOXC1 SE (SSE245). *n* = 3 independent experiments. **(d)** mRNA levels of FOXC1 upon JQ1 treatment. Data represent Mean ± SEM of 3 independent experiments. **(e)** spheroid growth of indicated cells with or without JQ1 treatment. *n* = 107 (DMSO), 169 (0.3 μM JQ1), 62 (1 μM JQ1) of MDA-MB-231 spheroids examined over 3 independent experiments. *n* = 93 (DMSO), 118 (0.3 μM JQ1), 179 (1 μM JQ1) of MCF10-DCIS spheroids examined over 3 independent experiments. *n* = 139 (DMSO), 104 (0.3 μM JQ1), 108 (1 μM JQ1) of BT549 spheroids examined over 3 independent experiments. Data are represented as mean ± SEM in **a**, **b**,

**c**, **d** and **e**. *P*-values calculated by one-sided Student's *t*-test are indicated in **a**, **b** and **c**. *p*-values by two-sided Student's *t*-test are indicated in **d** and **e**. Source data are provided as a Source Data file.

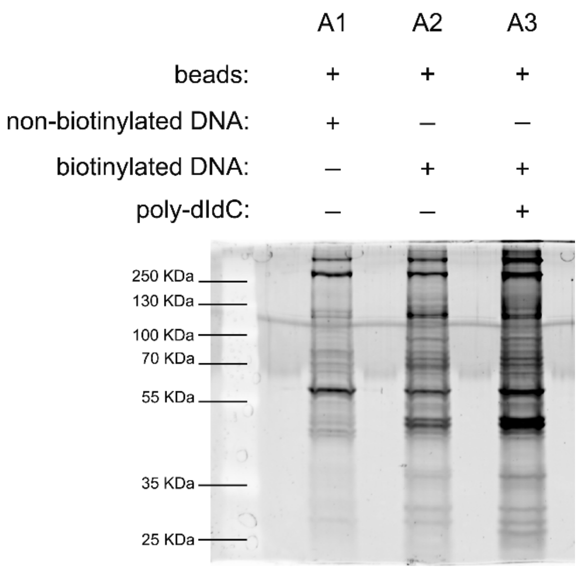


**Supplementary Fig. 8. SDS-PAGE of DNA pull down assay samples.** Experiments were repeated twice independently with similar results.

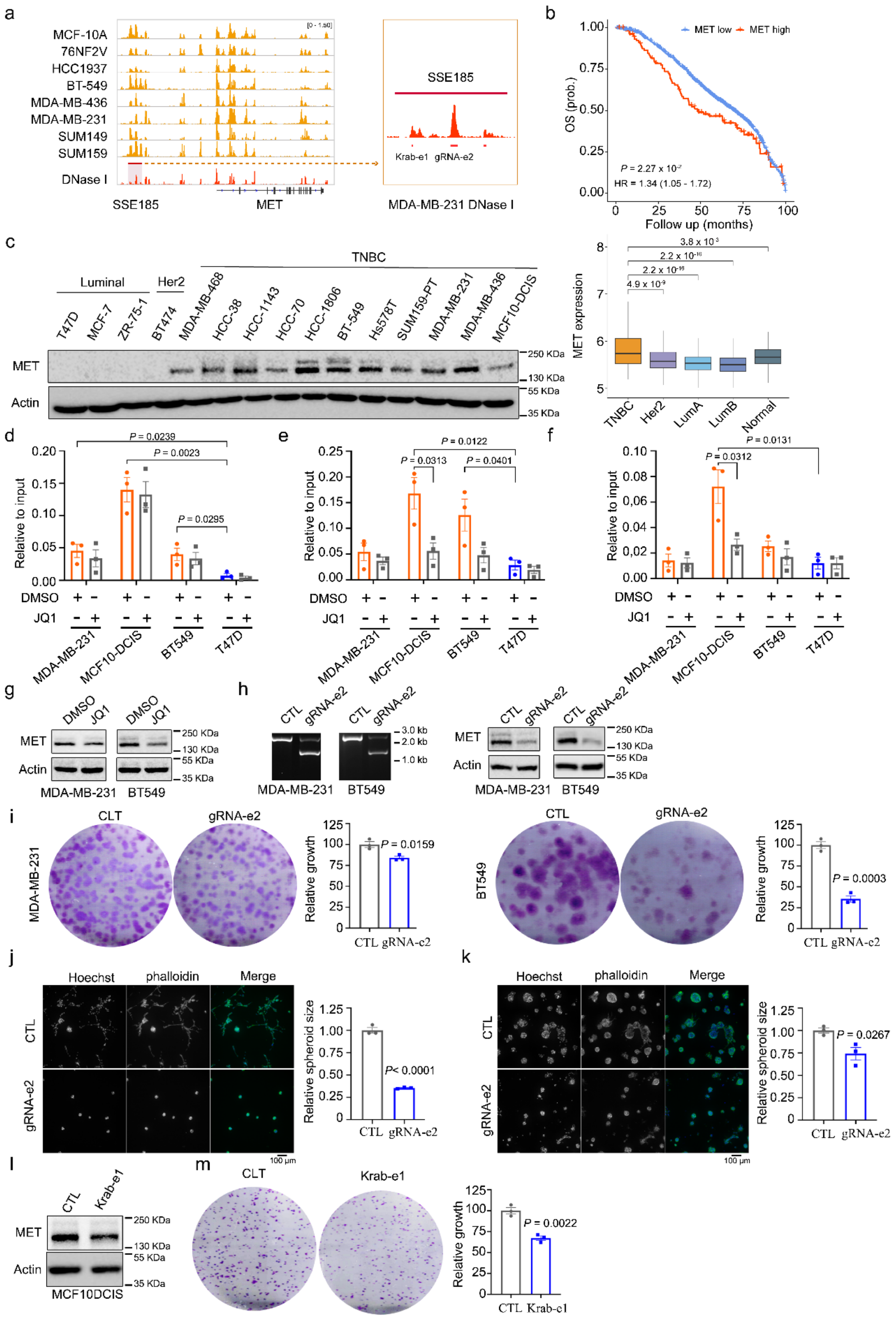

**Supplementary Fig. 9. Functional importance of MET super-enhancer in TNBC. (a)** Schematic illustrates the SE of MET. **(b)** Higher expression of MET is associated with worse prognosis of breast cancer patients in the METABRIC cohort. The statistical

significance was calculated by a log-rank test (one-sided). Higher MET expression levels in TNBC samples (*n* = 327) compared to other subtypes of breast cancer (two-sided Wilcoxon signed-rank tests, Her2 *n* = 237, LumA *n* = 706, LumB *n* = 484, and Normal-like *n* = 199). The boxes represent the 25th percentile, median, and 75th percentile, whiskers were extended to the furthest value that is no more than 1.5 times the inter-quartile range. (**c**) Immunoblotting detection of MET expression in a panel of breast cancer cell lines. (**d-f**) H3K27ac (**d**), Brd4 (**e**) and P300 (**f**) ChIP-qPCR of indicated cell lines using primers amplifying e2 of MET SE (SSE185). Data represent Mean ± SEM of 3 independent experiments. (**g**) Immunoblotting detection of MET expression in MDA-MB-231 and BT549 cells treated with JQ1 or DMSO. (**h**) Left, PCR detection of Crispr/Cas9-mediated deletion of e2 of MET SE. Right, immunoblotting detection of MET expression in MDA-MB-231and BT549 cells with deletion of e2. (**i**) Clonogenic assay of MDA-MB-231 and BT549 cells with or without deletion of e2. Bar graphs show the quantification of clonogenic proliferation, data represent Mean ± SEM of 3 independent experiments. (**j,k**) Phalloidin and Hoechst staining of MDA-MB-231and BT549 spheroids. Bar graphs show the relative spheroid size. Data represent Mean ± SEM of 3 independent experiments. **(l)** MET expression levels in MCF10-DCIS with or without KRAB-dCas9-mediated repression of e1. **(m)** Clonogenic assay of MCF10-DCIS cells. Data represent Mean ± SEM of 3 independent experiments. Experiments were repeated twice independently with similar results in **c**, **g**-**l**. *P*-values calculated by two-sided Student's *t*-test are indicated in **d**, **e**, **f**, **i**, **j**, **k** and **m**. Source data are provided as a Source Data file.

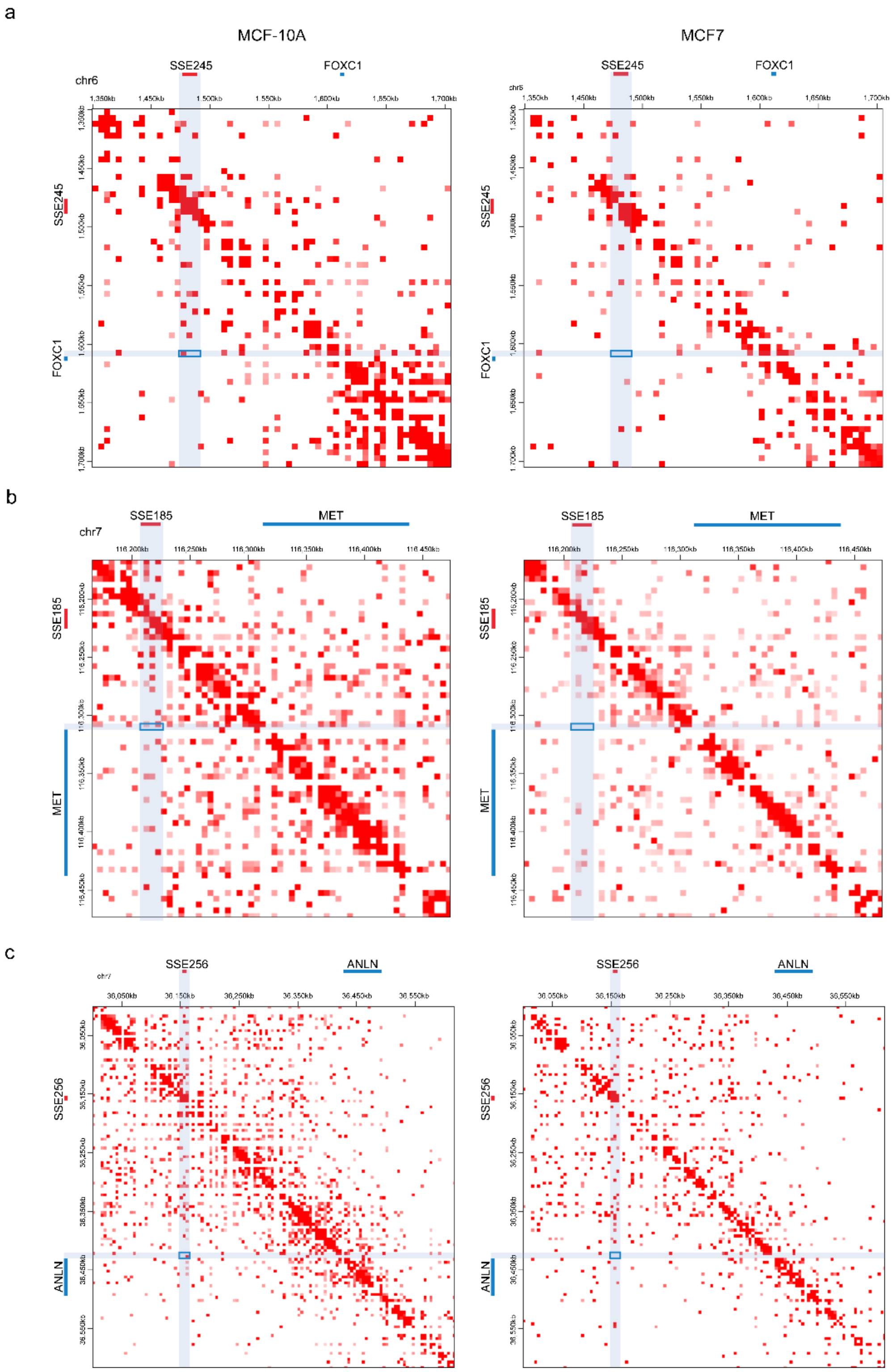


**Supplementary Fig. 10. The contacts between the SEs and the promoters.** Hi-C data demonstrated the contacts between the SEs and the promoters (5kb upstream of the TSSs) of FOXC1 **(a)**, MET **(b)** and ANLN **(c)** in MCF-10A but not in MCF7 cells.

**Supplementary Table 1. A summary of public datasets analyzed in the study**

Cell line sample

| **Subtype** | **Cell line** | **H3K27ac** | **Input** | **H3K4me1** | **H3K4me3** | **H3K27me3** | **RRBS** | **RNA-seq** | **BRD4 DMSO** | **BRD4 JQ1 treated** | **H3K27ac DMSO** | **H3K27ac JQ1 treated** | **DNase-seq** | **CTCF** | **Hi-C** |
|---|---|---|---|---|---|---|---|---|---|---|---|---|---|---|---|
| TNBC | BT549 | GSM1589476 (GSE69107) | GSM1589475 (GSE69107) | | | | SRR8633793 (PRJNA523380) | SRR8616197 (PRJNA523380) | | | | | | | |
| TNBC | CAL 51 | GSM1693015 (GSE38548) | GSM1693027 (GSE38548) | | | | SRR8633844 (PRJNA523380) | SRR8615264 (PRJNA523380) | | | | | | | |
| TN, immortalized breast line | MCF-10A | GSM2258704 (GSE85158) | GSM2258720 (GSE85158) | GSM2258710 (GSE85158) | GSM2258712 (GSE85158) | GSM2258706 (GSE85158) | | | | | | | | GSM2599084 (GSE98551) | GSM1631184 (GSE66733) |
| TN, immortalized breast line | 76NF2V | GSM2258686 (GSE85158) | GSM2258702 (GSE85158) | GSM2258692 (GSE85158) | GSM2258694 (GSE85158) | GSM2258688 (GSE85158) | | | | | | | | | |
| TNBC | MDA-MB-468 | GSM1589470 (GSE69107) | GSM1589471 (GSE69107) | GSM2258890 (GSE85158) | GSM2258892 (GSE85158) | GSM2258886 (GSE85158) | SRR8633350 (PRJNA523380) | SRR8615578 (PRJNA523380) | | | | | | | |
| TNBC | MDA-MB-436 | GSM2258866 (GSE85158) | GSM2258882 (GSE85158) | GSM2258872 (GSE85158) | GSM2258874 (GSE85158) | GSM2258868 (GSE85158) | SRR8633370 (PRJNA523380) | SRR8615584 (PRJNA523380) | GSM1842700 (GSE63584) | GSM1842701 (GSE63584) | | | | | |

| | | | | | | | | | | | | | | | |
|---|---|---|---|---|---|---|---|---|---|---|---|---|---|---|---|
| TNBC | MDA-MB-231 | GSM2258848 (GSE85158) | GSM2258864 (GSE85158) | GSM2258854 (GSE85158) | GSM2258856 (GSE85158) | GSM2258850 (GSE85158) | SRR8633711 (PRJNA523380) | SRR8615767 (PRJNA523380) | | | | | GSM2242136 (GSE84579) | | |
| TNBC | HCC1937 | GSM2258902 (GSE85158) | GSM2258918 (GSE85158) | GSM2258908 (GSE85158) | GSM2258910 (GSE85158) | GSM2258904 (GSE85158) | SRR8634002 (PRJNA523380) | SRR8616179 (PRJNA523380) | | | | | | | |
| TNBC | SUM149 | GSM1842713 (GSE63584) | GSM1842715 (GSE63584) | | | | | | GSM1842711 (GSE63584) | GSM1842712 (GSE63584) | GSM1842713 (GSE63584) | GSM1842714 (GSE63584 ) | | | |
| TNBC | SUM159 | GSM1553142 (GSE63584) | GSM1553140 (GSE63584) | GSM2330569 (GSE87424) | GSM1122666 (GSE46073) | | | | GSM1553139 (GSE63584) | GSM1553141 (GSE63584) | GSM1553142 (GSE63584) | GSM1553143 (GSE63584) | | | |
| TNBC | HCC1395 | | | | | | | | GSM1842697 (GSE63584) | GSM1842698 (GSE63584) | | | | | |
| TNBC | SUM1315 | | | | | | | | GSM1842703 (GSE63584) | GSM1842704 (GSE63584) | | | | | |
| non-TNBC | MCF-7 | GSM2258722 (GSE85158) | GSM2258738 (GSE85158) | GSM2258728 (GSE85158) | GSM2258730 (GSE85158) | GSM2258724 (GSE85158) | SRR8633964 (PRJNA523380) | SRR8615758 (PRJNA523380) | | | | | | GSM1817665 (GSE70764) | GSM1631185 (GSE66733) |
| non-TNBC | ZR-75-1 | GSM1589472 (GSE69107) | GSM1589477 (GSE69107) | GSM2258746 (GSE85158) | GSM2258748 (GSE85158) | GSM2258742 (GSE85158) | SRR8634106 (PRJNA523380) | SRR8618301 (PRJNA523380) | | | | | | | |

| | | | | | | | | |
|---|---|---|---|---|---|---|---|---|
| non-TNBC | MDA-MB-361 | GSM2258758 (GSE85158) | GSM2258774 (GSE85158) | GSM2258764 (GSE85158) | GSM2258766 (GSE85158) | GSM2258760 (GSE85158) | SRR8633710 (PRJNA523380) | SRR8615581 (PRJNA523380) |
| non-TNBC | UACC812 | GSM2258776 (GSE85158) | GSM2258792 (GSE85158) | GSM2258782 (GSE85158) | GSM2258784 (GSE85158) | GSM2258778 (GSE85158) | SRR8633265 (PRJNA523380) | SRR8615338 (PRJNA523380) |
| non-TNBC | SKBR3 | GSM2258794 (GSE85158) | GSM2258810 (GSE85158) | GSM2258800 (GSE85158) | GSM2258802 (GSE85158) | GSM2258796 (GSE85158) | SRR8633679 (PRJNA523380) | SRR8615677 (PRJNA523380) |
| non-TNBC | AU565 | GSM2258812 (GSE85158) | GSM2258828 (GSE85158) | GSM2258818 (GSE85158) | GSM2258820 (GSE85158) | GSM2258814 (GSE85158) | SRR8633295 (PRJNA523380) | SRR8615774 (PRJNA523380) |
| non-TNBC | HCC1954 | GSM2258830 (GSE85158) | GSM2258846 (GSE85158) | GSM2258836 (GSE85158) | GSM2258838 (GSE85158) | GSM2258832 (GSE85158) | SRR8634001 (PRJNA523380) | SRR8616174 (PRJNA523380) |
| non-TNBC | T-47-D | GSM1589474 (GSE69107) | GSM1589473 (GSE69107) | GSM659794 (GSE26831) | GSM2131187 (GSE80592) | GSM1541450 (GSE63109) | SRR8633760 (PRJNA523380) | SRR8615812 (PRJNA523380) |
| non-TNBC | BT474 | ERR908366 (PRJEB9547) | ERR908350 (PRJEB9547) | | | | SRR8634125 (PRJNA523380) | SRR8616195 (PRJNA523380) |

Patient sample

| Cohort | No. of samples | Resource |
|---|---|---|
| TCGA-BRCA | 720 | TCGA |
| METABRIC | 1953 | METABRIC |
| GSE5327 | 58 | Gene Expression Omnibus |
| GSE1456 | 159 | Gene Expression Omnibus |
| GSE2034 | 286 | Gene Expression Omnibus |
| GSE2990 | 102 | Gene Expression Omnibus |
| GSE11121 | 200 | Gene Expression Omnibus |
| GSE3494 | 251 | Gene Expression Omnibus |
| GSE7390 | 198 | Gene Expression Omnibus |
| GSE12276 | 204 | Gene Expression Omnibus |

Samples with molecular subtyping labels and corresponding clinical information were kept for analyses.

**Supplementary Table 2. The number of typical enhancers and super-enhancers**

| cell line | total NO. of cis-regulatory elements | NO. of super-enhancers | NO. of typical enhancers |
|---|---|---|---|
| AU565 | 40293 | 2338 | 37955 |
| BT474 | 25458 | 1362 | 24096 |
| BT549 | 17716 | 1362 | 16354 |
| CAL51 | 15484 | 772 | 14712 |
| HCC-1937 | 37288 | 1924 | 35364 |
| HCC-1954 | 34225 | 1648 | 32577 |
| MCF-10A | 42164 | 2791 | 39373 |
| MCF-7 | 12684 | 326 | 12358 |
| MDA-MB-231 | 33892 | 1520 | 32372 |
| MDA-MB-361 | 34073 | 1205 | 32868 |
| MDA-MB-436 | 43989 | 2771 | 41218 |
| MDA-MB-468 | 27582 | 1610 | 25972 |
| 76NF2V | 58314 | 3156 | 55158 |
| SKBR3 | 28254 | 1920 | 26334 |
| SUM149 | 34057 | 2080 | 31977 |
| SUM159 | 52558 | 3305 | 49253 |
| T-47-D | 29704 | 1578 | 28126 |
| UACC812 | 30759 | 1631 | 29128 |
| ZR-75-1 | 33264 | 1382 | 31882 |

**Supplementary Table 3. Correlation of FOXC1 expression with clinicopathology parameters, and summary of FOXC1 staining intensity**

| Parameter | FOXC1+ | FOXC1- | *P (Chi-square test )* |
|---|---|---|---|
| TNBC | 26 | 22 | *P* = 2.91e-08 |
| non-TNBC | 11 | 91 | |
| Grade 1&2 | 13 | 75 | *P* = 9.72e-05 |
| Grade 3 | 23 | 26 | |
| Ki67 <15% | 32 | 60 | *P* = 6.14e-04 |
| Ki67 ≥15% | 5 | 53 | |
| TILs <50% | 11 | 85 | *P* = 1.54e-06 |
| TILs ≥50% | 26 | 28 | |

TILs, tumor-infiltrating lymphocytes

| Specimen type | Diagnosis | Mean staining intensity of FOXC1 (0-3) |
|---|---|---|
| Surgical specimen | TNBC | 2.16 |
| | Basal-like TNBC | 2.6 |
| | LN metastasis | 2 |
| | non-TNBC | 0.3 |
| Tissue microarray | TNBC | 1.01 |
| | Basal-like TNBC | 2.38 |
| | non-TNBC | 0.22 |

**Supplementary Table 4. List of primers and gRNAs**

| **e1 of FOXC1 deletion pair** | Target | PAM | DNA coordinates (Human hg19) |
|---|---|---|---|
| e1del_gRNA1 | GTCCATCTCTGGTATATCTC | TGG | Chr6: 1481010-1481032(+) |
| e1del_gRNA2 | GCATGGATTGGTGTGCGACA | AGG | Chr6: 1482213-1482235(+) |

| **e2 of FOXC1 deletion pair** | Target | PAM | DNA coordinates (Human hg19) |
|---|---|---|---|
| e2del_gRNA1 | TTGACTTGAGGAAGGCTCAA | AGG | Chr6: 1485624-1485646(+) |
| e2del_gRNA2 | TAACAGATGTACCGCTGTCC | TGG | Chr6:1486531-1486553(+) |

| **e2 of MET deletion pair** | Target | PAM | DNA coordinates (Human hg19) |
|---|---|---|---|
| e2del_gRNA1 | ATGTGGCTGTCAGCATAAGT | AGG | Chr7:116216566-116216588(+) |
| e2del_gRNA2 | GTTTCGCGCATGCTGTTGCC | TGG | Chr7:116217641-116217663(-) |

| **Super-enhancer of ANLN deletion pair** | Target | PAM | DNA coordinates (Human hg19) |
|---|---|---|---|
| ANLN_SE_del_gRNA1 | GTGTTGACAGTGGATGACTG | AGG | chr7:36155147-36155169(+) |
| ANLN_SE_del_gRNA2 | ATTTGGTAGAAGGAGGCTAG | AGG | chr7:36155774-36155796(-) |

| **PCR primers to detect the deletion e1 of FOXC1** | | DNA coordinates |
|---|---|---|
| e1del_F | TCCTCTACAACCAGGGCAGT | Chr6: 1480725-1480744 (+) |
| e1del_R | TTCCTCACTCATCGTCCAGC | Chr6: 1482595-1482614 (-) |

| **PCR primers to detect the deletion e2 of FOXC1** | | DNA coordinates |
|---|---|---|
| e2del_F | CACTGCAAGCCCACTTGAAC | Chr6: 1485239- 1485258 (+) |
| e2del_R | AGCAAGGAAGGATTCTGGGC | Chr6: 1486973- 1486992 (-) |

| PCR primers to detect the deletion e2 of MET | | | DNA coordinates |
|---|---|---|---|
| e2del_F | AGGGCAGAGTAGTCAAGTGG | | Chr7: 116216089- 116216108 (+) |
| e2del_R | TGTGGTTATCTGTAGCTGGCTC | | Chr7: 116218311- 116218332 (-) |

| PCR primers to detect the deletion ANLN SE | | | DNA coordinates |
|---|---|---|---|
| SEdel_F | GCAACAGCTCTGAATGATTGGT | | chr7:36154347-36154368(+) |
| SEdel_R | TCTTTACCGCTAATGGCCCG | | chr7:36155897-36155916(-) |

| knockout of ANLN | Target | PAM | DNA coordinates (Human hg19) |
|---|---|---|---|
| ANLN_gRNA1 | TGCTGGAGCGAACCCGTGCC | AGG | Chr7: 36435879-36435901 (+) |
| ANLN_gRNA2 | TCTCTGAAGATTCTCTCGCC | TGG | Chr7: 36435897-36435919 (-) |

| dCas9-Krab gRNA of e1 of MET | Target | PAM | DNA coordinates (Human hg19) |
|---|---|---|---|
| Krab-e1 gRNA | CCAGATTCTCTCATCTTCCG | TGG | Chr7:116209959-116209981(+) |

| Chip-qPCR primers of e1 of FOXC1 | | | DNA coordinates (Human hg19) |
|---|---|---|---|
| e1-Chip-qPCR-F | AGGGGTCTATCCACATGGCT | | Chr6:1481807 -1481826 (+) |
| e1-Chip-qPCR-R | AGCATCAGGTCAGGGAATGC | | Chr6: 1481934-1481953 (-) |

| Chip-qPCR primers of e2 of FOXC1 | | | DNA coordinates (Human hg19) |
|---|---|---|---|
| e2-Chip-qPCR-F | AGTCAGCTCCTGTGAACAGC | | Chr6: 1486069- 1486088(+) |
| e2-Chip-qPCR-R | CCAGCTGTCAAGGTCACCTT | | Chr6: 1486187- 1486206(-) |

| Chip-qPCR primers of e2 of MET | | | DNA coordinates (Human hg19) |
|---|---|---|---|

| | | |
|---|---|---|
| e2-Chip-qPCR-F | CCACTTAATAACTGTGCAGGGC | Chr7: 116217198-116217219(+) |
| e2-Chip-qPCR-R | CTCGCAGTTCTATGGGGGTG | Chr7: 116217298-116217317 (-) |

| **Chip-qPCR primers of e1 of ANLN** | | DNA coordinates (Human hg19) |
|---|---|---|
| e1-Chip-qPCR-F | TCTCCCACCCACTCTGTAGG | Chr7: 36155298- 36155317(+) |
| e1-Chip-qPCR-R | TCTCTGGGTGTCCCTTGTGA | Chr7: 36155417- 36155436(-) |

| **PCR primers of biotinated-DNA of e1 of FOXC1-SE** | |
|---|---|
| DNA pulldown assay-e1-F | biotinated-GAGACAGGGTCTCACTCTGT |
| DNA pulldown assay-e1-R | CGCACACCAATCCATGCAAT |

| **Primers for cloning individual enhancers** | Sequence | DNA coordinates (hg19) | Fragments or Constituent Enhancers of FOXC1-SE |
|---|---|---|---|
| fragment containing e1-F | TCCTCTACAACCAGGGCAGT | Chr6: 1480725-1480744 (+) | fragments containing e1 |
| fragment containing e1-R | TTCCTCACTCATCGTCCAGC | Chr6: 1482595-1482614 (-) | |
| (+)MluI-e1-F | CTGTT acgcgt **GGACCACAGGCATGCACCAC** | Chr6: 1481188-1481207(+) | (+) e1 |
| (+)XhoI-e1-R | ACGG ctcgag **ATGCACACATCAGCTTTTCAAGGC** | Chr6: 1482235-1482258 (-) | |
| (-)MluI-e1-F | CTGTT acgcgt **ATGCACACATCAGCTTTTCAAGGC** | Chr6: 1482235-1482258 (-) | (-) e1 |
| (-)XhoI-e1-R | ACGG ctcgag **GGACCACAGGCATGCACCAC** | Chr6: 1481188-1481207(+) | |
| fragment containing e2-F | CACTGCAAGCCCACTTGAAC | Chr6: 1485239-1485258 (+) | fragments containing e2 |
| fragment containing e2-R | AGCAAGGAAGGATTCTGGGC | Chr6: 1486973-1486992 (-) | |

| | | | |
|---|---|---|---|
| (+)MluI-e2-F | CTGTT acgcgt **AATCCACATTCAGTAGGAATCAT** | chr6: 1485648-1485670 (+) | (+) e2 |
| (+)XhoI-e2-R | ACGG ctcgag **GTTCACACCCAGAGTTCAGA** | chr6: 1486483-1486502(-) | |
| (-)MluI-e2-F | CTGTT acgcgt **GTTCACACCCAGAGTTCAGA** | chr6: 1486483-1486502(-) | (-) e2 |
| (-)XhoI-e2-R | ACGG ctcgag **AATCCACATTCAGTAGGAATCAT** | chr6: 1485648-1485670 (+) | |
| | | | |
| fragment containing e3-F | TGGGACCAAGAGCATGTGAT | chr6: 1477321-1477340(+) | fragments containing e3 |
| fragment containing e3-R | AGTTCGCGTGTAGAAGTCAGC | chr6: 1478129-1478149(-) | |
| (+)MluI-e3-F | CTGTT acgcgt **CAAATGCTCATTTTCAAATGTTGA** | chr6: 1477621-1477644(+) | (+) e3 |
| (+)XhoI-e3-R | ACGG ctcgag **GAAGTCAGCCCACCCAGGCT** | chr6: 1478118-1478137(-) | |
| (-)MluI-e3-F | CTGTT acgcgt **GAAGTCAGCCCACCCAGGCT** | chr6: 1478118-1478137(-) | (-) e3 |
| (-)XhoI-e3-R | ACGG ctcgag **CAAATGCTCATTTTCAAATGTTGA** | chr6: 1477621-1477644(+) | |
| | | | |
| fragment containing e4-F | TTACCGTGAGGAGGCACCTAT | chr6: 1482966-1482986(+) | fragments containing e4 |
| fragment containing e4-R | CACATTTGTTCATGCTCCCTGG | chr6: 1483893-1483914(-) | |
| (+)MluI-e4-F | CTGTT acgcgt **TTACTATTTTTTGAGCATTTACTGTGT** | chr6: 1482999-483025(+) | (+) e4 |
| (+)XhoI-e4-R | ACGG ctcgag **TGAGCTACTGCATCCGGCC** | chr6: 1483329-1483347(-) | |
| (-)MluI-e4-F | CTGTT acgcgt **TGAGCTACTGCATCCGGCC** | chr6: 1483329-1483347(-) | (-) e4 |

| | | |
|---|---|---|
| (-)XhoI-e4-R | ACGG ctcgag **TTACTATTTTTTGAGCATTTACTGTGT** | chr6: 1482999-483025(+) |

| **FOXC1-shRNA** | |
|---|---|
| shFOXC Top | CCGGGAGCTTTCGTCTACGACTGTACTCGAGTACAGTCGTAGACGAAAGCTCTTTTT |
| shFOXC bottom | AATTAAAAAGAGCTTTCGTCTACGACTGTACTCGAGTACAGTCGTAGACGAAAGCTC |
