# Supplementary material for: Defining super-enhancer landscape in triple-negative breast cancer by multiomic profiling
Source: Nat Commun. 2021 Apr 14;12:2242. doi: 10.1038/s41467-021-22445-0 (PMC8046763; doi:10.1038/s41467-021-22445-0)
Supplement: Supplementary file 2 — Description of Additional Supplementary Files [file 41467_2021_22445_MOESM2_ESM.pdf]

Description of Additional Supplementary Files

File Name: Supplementary Data 1.

Description: The detailed statistical quantification of SE similarities between cell lines in the association network.

File Name: Supplementary Data 2.

Description: TNBC-specific super-enhancers and predicted target genes.

File Name: Supplementary Data 3.

Description: Predicted TFs that potentially bind to the five constituent enhancers of SSE245.

File Name: Supplementary Data 4.

Description: Mass spectrum analysis.
