# Supplementary material for: Defining super-enhancer landscape in triple-negative breast cancer by multiomic profiling
Source: Nat Commun. 2021 Apr 14;12:2242. doi: 10.1038/s41467-021-22445-0 (PMC8046763; doi:10.1038/s41467-021-22445-0)
Supplement: Supplementary file 7 — Reporting Summary [file 41467_2021_22445_MOESM7_ESM.pdf]

Corresponding author(s): Y Rebecca Chin, Xin Wang

Last updated by author(s): Feb 4, 2021

Reporting Summary

Nature Research wishes to improve the reproducibility of the work that we publish. This form provides structure for consistency and transparency in reporting. For further information on Nature Research policies, see our Editorial Policies and the Editorial Policy Checklist.

Statistics

For all statistical analyses, confirm that the following items are present in the figure legend, table legend, main text, or Methods section.

n/a	Confirmed	
☐	☒	The exact sample size (*n*) for each experimental group/condition, given as a discrete number and unit of measurement
☐	☒	A statement on whether measurements were taken from distinct samples or whether the same sample was measured repeatedly
☐	☒	The statistical test(s) used AND whether they are one- or two-sided<br>*Only common tests should be described solely by name; describe more complex techniques in the Methods section.*
☒	☐	A description of all covariates tested
☐	☒	A description of any assumptions or corrections, such as tests of normality and adjustment for multiple comparisons
☐	☒	A full description of the statistical parameters including central tendency (e.g. means) or other basic estimates (e.g. regression coefficient) AND variation (e.g. standard deviation) or associated estimates of uncertainty (e.g. confidence intervals)
☐	☒	For null hypothesis testing, the test statistic (e.g. *F*, *t*, *r*) with confidence intervals, effect sizes, degrees of freedom and *P* value noted<br>*Give P values as exact values whenever suitable.*
☒	☐	For Bayesian analysis, information on the choice of priors and Markov chain Monte Carlo settings
☒	☐	For hierarchical and complex designs, identification of the appropriate level for tests and full reporting of outcomes
☒	☐	Estimates of effect sizes (e.g. Cohen's *d*, Pearson's *r*), indicating how they were calculated

Our web collection on statistics for biologists contains articles on many of the points above.

Software and code

Policy information about availability of computer code

Data collection

No software was used.

Data analysis

We have specified all open source software in the Methods section. The code used for the entire data analysis process is freely available on github (https://github.com/CityUHK-CompBio/TNBCSE_code).

1. ChIP-seq analysis
H3K27ac, H3K4me1, H3K4me3, H3K27me3, CTCF and BRD4 ChIP-seq reads were mapped to the human reference genome (UCSC hg19 genome build) using Bowtie (version 1.2.2), retaining only uniquely mapped reads for downstream analyses. For each mark, coverage tracks were generated using 'deeptools' (version 3.3.0) and enrichment signals of histone modifications were scaled by Counts Per Million (or CPM) mapped reads. H3K27ac enrichment regions were identified using a two-state hidden Markov model (ChromHMM, version 1.17). The density of normalized mapped reads in SEs by Bamliquidator (version 1.3.8, https://github.com/BradnerLab/pipeline/wiki/bamliquidator).

2. RNA-seq analysis
Sequencing reads were mapped to the human reference genome (UCSC hg19 assembly) using STAR (version 2.7.1a) , where only uniquely mapped reads were retained for the subsequent analyses. Differential gene expression analysis was performed using the R package 'DESeq2' (version 1.26.0) or 'limma' (R package, version 3.32.2).

3. RRBS analysis
Reduced representation bisulfite sequencing (RRBS) raw reads were aligned to the human reference genome (UCSC hg19 assembly) using Bismark (v0.20), powered by bowtie2 (version 2.3.5), with default parameters. Based on the resulting BAM files, DNA methylation levels of SEs and promoter (+/- 2.5kb of TSS) regions of corresponding genes were calculated using the R package 'methylKit' (version 1.2.10).

4. Regulatory network inference and master regulator analysis
We inferred a regulatory network to investigate the relationships between SE regulated TFs and potential targets by integrative analysis of gene expression profiles of breast cancer patient samples in the TCGA-BRCA dataset. TFs regulated by TNBC-specific SEs that are notably up-regulated (H3K27ac log2 fold enrichment > 1, BH-adjusted P < 0.05 & gene expression log2 fold change> 1, BH-adjusted P < 0.05) in TNBC-subtype patients compared to the others, were prioritized as potential regulators. Genes differentially expressed between TNBC and non-TNBC tumors (log2 fold change> 0.5, BH-adjusted P < 0.05) were considered as potential targets of the TFs. The TFs and potential targets were integrated for network inference using the 'RTN' package (version 1.12.0). Master regulator analysis was performed using a hypergeometric test of overrepresentation of each TF's predicted regulon for a specific gene signature.

For manuscripts utilizing custom algorithms or software that are central to the research but not yet described in published literature, software must be made available to editors and reviewers. We strongly encourage code deposition in a community repository (e.g. GitHub). See the Nature Research guidelines for submitting code & software for further information.

Data

Policy information about availability of data

All manuscripts must include a data availability statement. This statement should provide the following information, where applicable:

- Accession codes, unique identifiers, or web links for publicly available datasets
- A list of figures that have associated raw data
- A description of any restrictions on data availability

ChIP-seq data (with corresponding input data) for H3K27ac (19 cell lines), H3K4me1 (15 cell lines), H3K4me3 (15 cell lines), H3K27me3 (14 cell lines), CTCF (2 cell lines) and BRD4 (5 cell lines, before and after JQ1 treated) markers, DNase-seq (2 cell lines) used in this study are available in Gene Expression Omnibus (GEO) and EMBL-EBI ArrayExpress under accession code GSE69107 [https://www.ncbi.nlm.nih.gov/geo/query/acc.cgi?acc=GSE69107], GSE38548 [https://www.ncbi.nlm.nih.gov/geo/query/acc.cgi?acc=GSE38548], GSE85158 [https://www.ncbi.nlm.nih.gov/geo/query/acc.cgi?acc=GSE85158], GSE63584 [https://www.ncbi.nlm.nih.gov/geo/query/acc.cgi?acc=GSE63584], GSE87424 [https://www.ncbi.nlm.nih.gov/geo/query/acc.cgi?acc=GSE87424], GSE46073 [https://www.ncbi.nlm.nih.gov/geo/query/acc.cgi?acc=GSE46073], GSE26831 [https://www.ncbi.nlm.nih.gov/geo/query/acc.cgi?acc=GSE26831], GSE80592 [https://www.ncbi.nlm.nih.gov/geo/query/acc.cgi?acc=GSE80594], GSE63109 [https://www.ncbi.nlm.nih.gov/geo/query/acc.cgi?acc=GSE63109], GSE84579 [https://www.ncbi.nlm.nih.gov/geo/query/acc.cgi?acc=GSE84579], GSE98551 [https://www.ncbi.nlm.nih.gov/geo/query/acc.cgi?acc=GSE98551], GSE70764 [https://www.ncbi.nlm.nih.gov/geo/query/acc.cgi?acc=GSE70764] and PRJEB9547 [https://www.ebi.ac.uk/ena/browser/view/PRJEB9547]. RNA-seq and reduced representation bisulfite sequencing (RRBS) data of 15 cell lines used in this study are available in EMBL-EBI ArrayExpress under accession code PRJNA523380 [https://www.ebi.ac.uk/ena/browser/view/PRJNA523380]. Hi-C data of MCF-10A and MCF-7 used in this study are available in GEO under accession code GSE66733 [https://www.ncbi.nlm.nih.gov/geo/query/acc.cgi?acc=GSE66733]. Gene expression profiles for primary breast cancer samples, together with molecular subtyping labels and corresponding clinical information used in this study are available in Firehose Broad GDAC portal (https://gdac.broadinstitute.org/, n = 720), Molecular Taxonomy of Breast Cancer International Consortium (METABRIC, https://www.cbioportal.org/, n = 1953) and GEO under accession code GSE5327 [https://www.ncbi.nlm.nih.gov/geo/query/acc.cgi?acc=GSE5327], GSE1456 [https://www.ncbi.nlm.nih.gov/geo/query/acc.cgi?acc=GSE1456], GSE2034 [https://www.ncbi.nlm.nih.gov/geo/query/acc.cgi?acc=gse2034], GSE2990 [https://www.ncbi.nlm.nih.gov/geo/query/acc.cgi?acc=GSE2990], GSE11121 [https://www.ncbi.nlm.nih.gov/geo/query/acc.cgi?acc=GSE11121], GSE3494 [https://www.ncbi.nlm.nih.gov/geo/query/acc.cgi?acc=GSE3494], GSE7390 [https://www.ncbi.nlm.nih.gov/geo/query/acc.cgi?acc=GSE7390] and GSE12276 [https://www.ncbi.nlm.nih.gov/geo/query/acc.cgi?acc=GSE12276]. More details about the data availability and curation were summarized in Supplementary Table 1. Source data are available as a Source Data file. Source data are provided with this paper.The remaining data are available within the Article, Supplementary Information or available from the authors upon request.

Field-specific reporting

Please select the one below that is the best fit for your research. If you are not sure, read the appropriate sections before making your selection.

☒ Life sciences ☐ Behavioural & social sciences ☐ Ecological, evolutionary & environmental sciences

For a reference copy of the document with all sections, see nature.com/documents/nr-reporting-summary-flat.pdf

Life sciences study design

All studies must disclose on these points even when the disclosure is negative.

Sample size	Sample size was chosen based on previous experiment done (Chin YR, 2014, PMID: 24838891; Chin YR, 2014, PMID: 24335962). Using power analysis, the anticipated means and standard error of means were included to determine the sample size that is expected to yield a power of approximately 80 percent using a p-value of 0.05.
Data exclusions	No.
Replication	Numbers of the experimental replication or the experiments that were performed independently for each specific result were indicated in the Figure Legends. All attempts at replication were successful.
Randomization	allocation of samples is random.
Blinding	blinded to group allocation.

Behavioural & social sciences study design

All studies must disclose on these points even when the disclosure is negative.

Study description	*Briefly describe the study type including whether data are quantitative, qualitative, or mixed-methods (e.g. qualitative cross-sectional, quantitative experimental, mixed-methods case study).*
Research sample	*State the research sample (e.g. Harvard university undergraduates, villagers in rural India) and provide relevant demographic information (e.g. age, sex) and indicate whether the sample is representative. Provide a rationale for the study sample chosen. For studies involving existing datasets, please describe the dataset and source.*
Sampling strategy	*Describe the sampling procedure (e.g. random, snowball, stratified, convenience). Describe the statistical methods that were used to predetermine sample size OR if no sample-size calculation was performed, describe how sample sizes were chosen and provide a rationale for why these sample sizes are sufficient. For qualitative data, please indicate whether data saturation was considered, and what criteria were used to decide that no further sampling was needed.*
Data collection	*Provide details about the data collection procedure, including the instruments or devices used to record the data (e.g. pen and paper, computer, eye tracker, video or audio equipment) whether anyone was present besides the participant(s) and the researcher, and whether the researcher was blind to experimental condition and/or the study hypothesis during data collection.*
Timing	*Indicate the start and stop dates of data collection. If there is a gap between collection periods, state the dates for each sample cohort.*
Data exclusions	*If no data were excluded from the analyses, state so OR if data were excluded, provide the exact number of exclusions and the rationale behind them, indicating whether exclusion criteria were pre-established.*
Non-participation	*State how many participants dropped out/declined participation and the reason(s) given OR provide response rate OR state that no participants dropped out/declined participation.*
Randomization	*If participants were not allocated into experimental groups, state so OR describe how participants were allocated to groups, and if allocation was not random, describe how covariates were controlled.*

Ecological, evolutionary & environmental sciences study design

All studies must disclose on these points even when the disclosure is negative.

Study description	*Briefly describe the study. For quantitative data include treatment factors and interactions, design structure (e.g. factorial, nested, hierarchical), nature and number of experimental units and replicates.*
Research sample	*Describe the research sample (e.g. a group of tagged Passer domesticus, all Stenocereus thurberi within Organ Pipe Cactus National Monument), and provide a rationale for the sample choice. When relevant, describe the organism taxa, source, sex, age range and any manipulations. State what population the sample is meant to represent when applicable. For studies involving existing datasets, describe the data and its source.*
Sampling strategy	*Note the sampling procedure. Describe the statistical methods that were used to predetermine sample size OR if no sample-size calculation was performed, describe how sample sizes were chosen and provide a rationale for why these sample sizes are sufficient.*
Data collection	*Describe the data collection procedure, including who recorded the data and how.*
Timing and spatial scale	*Indicate the start and stop dates of data collection, noting the frequency and periodicity of sampling and providing a rationale for these choices. If there is a gap between collection periods, state the dates for each sample cohort. Specify the spatial scale from which the data are taken*
Data exclusions	*If no data were excluded from the analyses, state so OR if data were excluded, describe the exclusions and the rationale behind them, indicating whether exclusion criteria were pre-established.*
Reproducibility	*Describe the measures taken to verify the reproducibility of experimental findings. For each experiment, note whether any attempts to repeat the experiment failed OR state that all attempts to repeat the experiment were successful.*
Randomization	*Describe how samples/organisms/participants were allocated into groups. If allocation was not random, describe how covariates were controlled. If this is not relevant to your study, explain why.*
Blinding	*Describe the extent of blinding used during data acquisition and analysis. If blinding was not possible, describe why OR explain why blinding was not relevant to your study.*

Did the study involve field work? ☐ Yes ☐ No

Field work, collection and transport

Field conditions	*Describe the study conditions for field work, providing relevant parameters (e.g. temperature, rainfall).*
Location	*State the location of the sampling or experiment, providing relevant parameters (e.g. latitude and longitude, elevation, water depth).*
Access & import/export	*Describe the efforts you have made to access habitats and to collect and import/export your samples in a responsible manner and in compliance with local, national and international laws, noting any permits that were obtained (give the name of the issuing authority, the date of issue, and any identifying information).*
Disturbance	*Describe any disturbance caused by the study and how it was minimized.*

Reporting for specific materials, systems and methods

We require information from authors about some types of materials, experimental systems and methods used in many studies. Here, indicate whether each material, system or method listed is relevant to your study. If you are not sure if a list item applies to your research, read the appropriate section before selecting a response.

Materials & experimental systems

n/a	Involved in the study	
☐	☒	Antibodies
☐	☒	Eukaryotic cell lines
☒	☐	Palaeontology and archaeology
☐	☒	Animals and other organisms
☐	☒	Human research participants
☒	☐	Clinical data
☒	☐	Dual use research of concern

Methods

n/a	Involved in the study	
☒	☐	ChIP-seq
☒	☐	Flow cytometry
☒	☐	MRI-based neuroimaging

Antibodies

Antibodies used

Anti-FOXC1 (#8758S) and anti-MET (#3127S) antibodies were obtained from Cell Signaling Technology. Anti-ANLN (Sigma, AMAB90660) and Anti-β-actin (A5441) antibody was purchased from Sigma-Aldrich. Horseradish peroxidase-conjugated anti-mouse and anti-rabbit immunoglobulin G antibodies (AP307P, AP308P) were purchased from Millipore. For immunohistochemistry staining, anti-FOXC1 (# ab223850) antibody was purchased from Abcam. Anti-Brd4 (Bethyl Laboratories, #A301-985a100), anti-P300 (Bethyl Laboratories, #A300-358a), and anti-H3K27ac (Active motif, #39685) antibodies were used for Chip-qPCR. All primary and secondary antibodies for immunoblotting were used at 1:1000 and 1:5000 dilution, respectively. For immunohistochemistry, FOXC1 antibody was diluted at 1:500. For ChIP-qPCR, 3 μg of anti-H3K27ac was used per sample, 5 μg of anti-P300 and anti-BRD4 were used per sample.

Validation

Anti-FOXC1 (#8758S) (Reactivity: Human, Mouse, Rat. Application: WB, IP) has been validated by manufacturer. 9 Citations. https://www.cellsignal.com/products/primary-antibodies/foxc1-d8a6-rabbit-mab/8758

anti-MET (#3127S) (Reactivity: Human, Mouse, Rat. Application: WB, IP) has been validated by manufacturer. 117 Citations. https://www.cellsignal.com/products/primary-antibodies/met-25h2-mouse-mab/3127?site-search-type=Products&N=4294956287&Ntt=anti-met+%28%233127s%29+&fromPage=plp&_requestid=1599553

anti-FOXC1 (# ab223850) (Reactivity: Human, Mouse, Rat. Application: IHC-P, IP, WB, Flow Cyt) has been validated by manufacturer. 2 Citations. https://www.abcam.com/foxc1-antibody-epr20678-ab223850.html

ANLN (Sigma, AMAB90660), (Reactivity: Human. Application: WB, ICC/IF) has been validated by manufacturer. https://www.sigmaaldrich.com/catalog/product/sigma/amab90660?lang=en&region=HK

Anti-β-Actin (Sigma, A5441), (Reactivity: Human, Mouse and so on. Application: WB, ICC/IF and so on) has been validated by manufacturer. https://www.sigmaaldrich.com/catalog/product/sigma/a5441?lang=en&region=HK&cm_sp=Insite-_-caContent_prodMerch_gruCrossEntropy-_-prodMerch10-1 . 6919 citations.

Anti-Brd4 (Bethyl Laboratories, #A301-985a100), (Reactivity: human, Mouse. Application: WB,IP, IHC.) has been validated by manufacturer. 45 citations. https://www.bethyl.com/product/A301-985A100/BRD4+Antibody . Citation for ChIP : Hong, S. H., & You, J. S. (2019). SOX9 is controlled by the BRD4 inhibitor JQ1 via multiple regulation mechanisms. Biochemical and biophysical research communications, 511(4), 746-752.

Anti-P300 (Bethyl Laboratories, #A300-358a), (Reactivity: human. Application: ChIP-seq, WB,IP, IHC.) has been validated by manufacturer. 14 citations. https://www.bethyl.com/product/A300-358A/p300+Antibody

Anti-H3K27ac (Active motif, #39685) (Reactivity: Human. Application: ChIP, ChIP-Seq, ICC/IF, WB) has been validated by manufacturer. 23 citations. https://www.activemotif.com/catalog/details/39685/histone-h3-acetyl-lys27-antibody-mab-clone-mabi-0309

Eukaryotic cell lines

Policy information about cell lines

Cell line source(s)	T47D, BT474, MCF-7, MDA-MB-436, MDA-MB-468, MDA-MB-231, Hs578T, ZR-75-1, HCC38, HCC1143, HCC70, HCC1806, BT-549 and HEK293T cells were obtained from ATCC. MCF10-DCIS and SUM159-PT cells were obtained from Kornelia Polyak (Harvard Medical School, USA).
Authentication	Cell lines obtained from ATCC were tested for authentication using short tandem repeat (STR) profiling. MCF10-DCIS and SUM159-PT cells were not authenticated.
Mycoplasma contamination	Cell lines were tested and free of mycoplasma contamination.
Commonly misidentified lines (See ICLAC register)	No commonly misidentified cell lines were used in this study.

Animals and other organisms

Policy information about studies involving animals; ARRIVE guidelines recommended for reporting animal research

Laboratory animals	Female Nude mice, 6-8 weeks old. Mice were housed in room temperature of 20-24 degree Celcius, with humidity of 30-70% and light/dark cycle of 12 hour/12 hour.
Wild animals	NO.
Field-collected samples	NO.
Ethics oversight	Female nude mice (6-8 weeks old) were purchased from the laboratory animal services centre, Chinese University of Hong Kong. All procedures were approved by the Animal Ethics Committees at City University of Hong Kong, and conform to the government guidelines for the care and maintenance of laboratory animals.

Note that full information on the approval of the study protocol must also be provided in the manuscript.

Human research participants

Policy information about studies involving human research participants

Population characteristics	breast cancer patients who underwent biopsy or surgery for resection at Queen Elizabeth Hospital, Hong Kong
Recruitment	N/A
Ethics oversight	Approved by Human Subjects Ethics Committees at City University of Hong Kong.

Note that full information on the approval of the study protocol must also be provided in the manuscript.
